# An interaction regression model for crop yield prediction

**DOI:** 10.1038/s41598-021-97221-7

**Published:** 2021-09-07

**Authors:** Javad Ansarifar, Lizhi Wang, Sotirios V. Archontoulis

**Affiliations:** 1 Department of Industrial and Manufacturing Systems Engineering, Iowa State University, Ames, IA 50011 USA; 2 Department of Agronomy, Iowa State University, Ames, IA 50011 USA

**Keywords:** Plant sciences, Machine learning

## Abstract

Crop yield prediction is crucial for global food security yet notoriously challenging due to multitudinous factors that jointly determine the yield, including genotype, environment, management, and their complex interactions. Integrating the power of optimization, machine learning, and agronomic insight, we present a new predictive model (referred to as the interaction regression model) for crop yield prediction, which has three salient properties. First, it achieved a relative root mean square error of 8% or less in three Midwest states (Illinois, Indiana, and Iowa) in the US for both corn and soybean yield prediction, outperforming state-of-the-art machine learning algorithms. Second, it identified about a dozen environment by management interactions for corn and soybean yield, some of which are consistent with conventional agronomic knowledge whereas some others interactions require additional analysis or experiment to prove or disprove. Third, it quantitatively dissected crop yield into contributions from weather, soil, management, and their interactions, allowing agronomists to pinpoint the factors that favorably or unfavorably affect the yield of a given location under a given weather and management scenario. The most significant contribution of the new prediction model is its capability to produce accurate prediction and explainable insights simultaneously. This was achieved by training the algorithm to select features and interactions that are spatially and temporally robust to balance prediction accuracy for the training data and generalizability to the test data.

## Introduction

Predicting crop yield is crucial to addressing emerging challenges in food security, particularly in an era of global climate change. Accurate yield predictions not only help farmers make informed economic and management decisions but also support famine prevention efforts. Underlying crop yield prediction is a fundamental research question in plant biology, which is to understand how plant phenotype is determined by genotype (G), environment (E), management (M), and their interactions (G $\times$ E $\times$ M)^1–6^. State-of-the-art crop yield prediction methods fall into three main categories: linear models, machine learning models, and crop models, which have complementary strengths and limitations. Linear models are explainable by quantifying the additive effect of each variable, but they often struggle to achieve high prediction accuracy due to the inability to capture the intrinsically nonlinear interactions among G, E, and M variables.

Machine learning models have been successfully used for crop yield prediction, including stepwise multiple linear regression^7^, random forest^8^, neural networks^9–11^, convolutional neural networks^12^, recurrent neural networks^13^, weighted histograms regression^14^, interaction based model^15^, and association rule mining and decision tree^16^. Most of these studies were based on environmental and managerial variables only, due to lack of publicly available genotype data at the state or national scale. Some studies^16–19^ explored the relationship between genotype and grain yield from regional yield trials from a plant breeding perspective, which would be hard to scale up to statewide or nationwide predictions. Many machine learning algorithms are scalable to large datasets and have reasonably high prediction accuracy. However, due to the black-box nature of these models, prediction accuracy is sensitive to model structure and parameter calibration, and it can prove difficult to explain why predictions are accurate or inaccurate.

Crop models are another type of nonlinear models, including APSIM^20^, DSSAT^21,22^, RZWQM^23^, and SWAP/WOFOST^24^, which build upon the physiological understanding of plant and soil processes to develop biologically meaningful non-linear equations to predict crop yield and other phenotypes. These models provide explicit (albeit complex) explanations of the interactions between traits and environmental conditions in different phases of the crop growth cycle. They also offer biological insights into causes of phenotypic variation^25^. Nevertheless, the collection of trait measurement data and calibration of model coefficients can be labor intensive and time consuming^26–29^, computation speed could be low^29^, and prediction accuracy may not be as high as some machine learning algorithms.

We propose a novel model, the interaction regression model, for crop yield prediction, which attempts to combine the strengths and avoid the limitations of the aforementioned approaches. At the core of this model lies a combinatorial optimization algorithm, which not only selects the most revealing E and M features but also detects their most pronounced interactions; the contributions of these features and interactions to the crop yield are then quantified with a multiple linear regression. To ensure the explainability of the results, we trained our algorithm to find features and interactions that are spatially and temporally robust, which means that they should be consistently predictive of crop yield across all counties in all years. As such, results from this model have the potential to propose biologically and agronomically insightful hypotheses on E $\times$ M interactions that can be validated experimentally. A similar concept of robust inference model in spatial–temporal models was presented in Santos and Erniel^30^. A measure of robustness was proposed in Nogueira et al.^31^, which was based on the number of overlapping features selected using different subsets of training data. In our approach, the robustness measure is defined as the average prediction performance in multiple validation datasets at different temporal and spatial spectra. As such, our robustness definition allowed the algorithm to strike a balance between prediction accuracy and generalizability.

The proposed model has demonstrated notable performance in a comprehensive case study, in which it was compared with eight other machine learning models to predict corn and soybean yield in 293 counties of the states of Illinois, Indiana, and Iowa from 2015 to 2018. Moreover, prediction performance with and without knowing weather during the growing season and temporal and spatial extrapolation performance of the proposed model in unseen counties were tested. The proposed model not only achieved a less than 8% relative root mean square error (RRMSE) for both corn and soybean in all three states, outperforming all other machine learning models in the case study, but also produced explainable insights. In particular, our model identified 11 E $\times$ M interactions for corn and 12 for soybean, and also dissected the total yield into contributions from weather, soil, management, and their interactions. To test the generalizability of the model in terms of both temporal and spatial extrapolation, we trained the model using historical data from two states up to 2017 and applied it to predict corn yield in a third state for 2018, and the resulting average RRMSE was less than 10%.

## Method

Let *X* denote the set of explanatory (including environment and management) variables and *y* the crop yield of a given county for a given year. We propose the interaction regression model to describe the relationship between *X* and *y* as follows.

1 $$\begin{aligned} \hat{y}_i =\beta _0 + \sum _{j \in {\mathcal {P}}} X_{i,j} \beta _j + \sum _{m \in \mathcal {M}} b_m Z_{i,m}, \quad \forall i \in \mathcal {N}, \end{aligned}$$

where,$\mathcal {N}$ is the set of sample observations (one sample per county per year), $\mathcal {P}$ is the set of explanatory variables, $\mathcal {M}$ is the set of interactions, $\hat{y}_i$ is predicted crop yield of sample *i*, $\beta _0$ is the intercept of crop yield, $\beta _j$ is the additive effect of variable *j*, $X_{i,j}$ is the explanatory variable *j* of sample *i*, $b_m$ is the effect of interaction *m*, and $Z_{i,m}$ is the interaction variable *m* of sample *i*.

Key to Eq. (1) is to decipher the interaction matrix *Z* from explanatory variables. We use a kernel-based approach to represent the interactions as

$$\begin{aligned} Z_{i,m} = \sum \limits _{k \in \mathcal {K}} \delta _{m,k} K_k(X_i), \end{aligned}$$

where, $K_k(\cdot )$ is the type *k* kernel function, $\mathcal {K}$ is the set of kernel functions that we use to describe nonlinear relationships between explanatory variables and crop yield, and $\delta _{m,k}$ is a binary variable indicating whether interaction *m* is best described by the type *k* kernel ($\delta _{m,k} = 1$) or not ($\delta _{m,k} = 0$).

**Figure 1:**
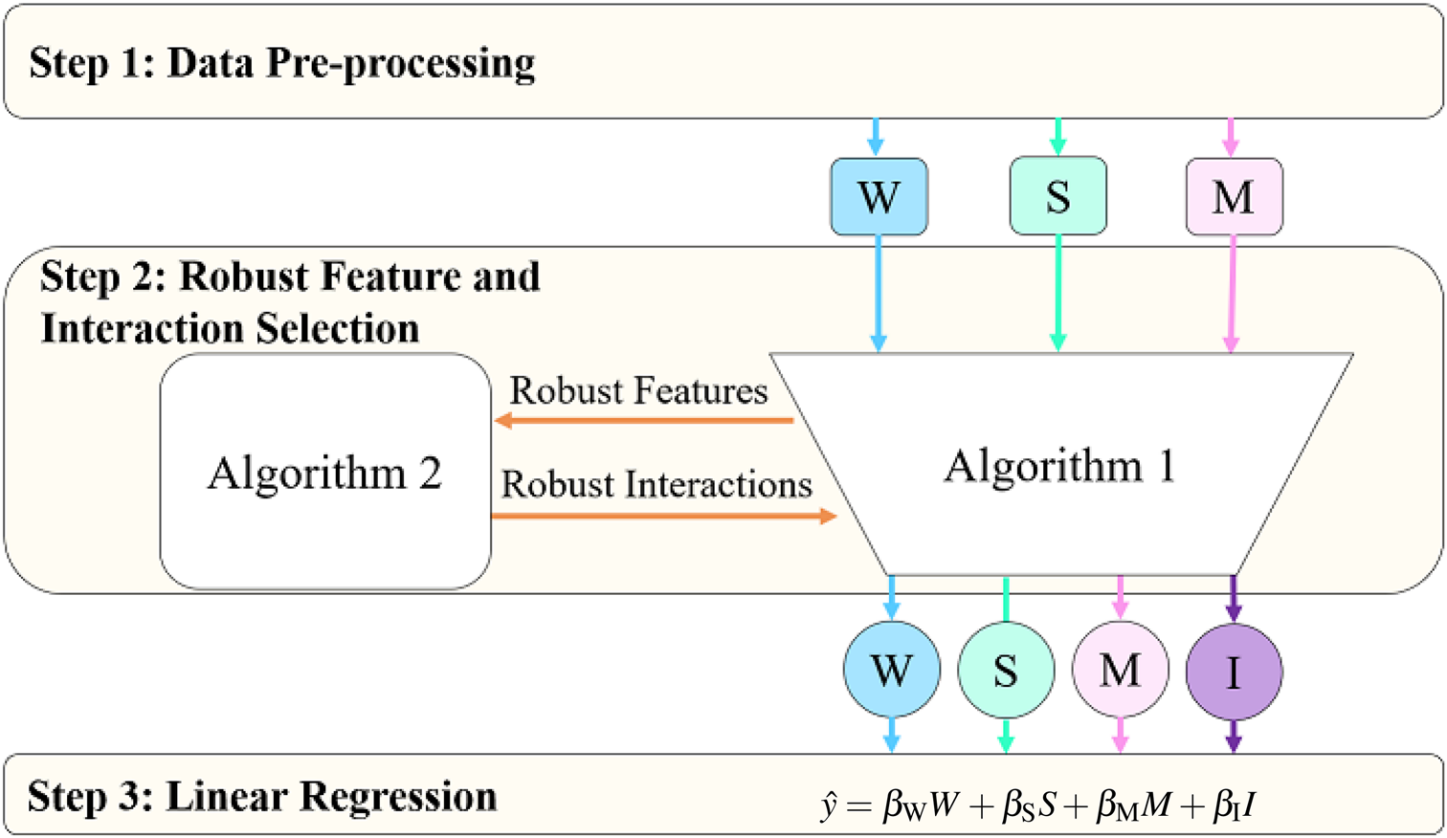
Illustration of the proposed interaction regression model for crop yield prediction. Step 1 is data pre-processing. In step 2, Algorithms 1 and 2 select robust features and interactions, which are then used in step 3 to predict the crop yield with a multiple linear regression model. Here, $\hat{y}$ is the predicted yield, $\beta _\text {W}$, $\beta _\text {S}$, and $\beta _\text {M}$ are, respectively, the additive effects of weather, soil, and management features, whereas $\beta _\text {I}$ is the effect of E × M interactions. This plot was created with Microsof PowerPoint (Version 16.0.12827.20200 32-bit).

In order to solve Eq. (1), we propose an approach that consists of three major steps: data pre-processing, robust feature and interaction selection, and linear regression, as illustrated in Fig. 1. Key elements of the three steps are summarized as follows.

### Step 1: Data pre-processing

We collected weather data from the Iowa Environmental Mesonet^32^, soil data from the Gridded Soil Survey Geographic Database^33^, and management and yield performance data from the National Agricultural Statistics Service^34^ for all 293 counties of the states of Illinois, Indiana, and Iowa from 1990 to 2018. Weather variables include precipitation (Prcp, mm), solar radiation (Srad, MJ m$^{-2}$), maximum temperature (Tmax, $^\circ$C), and minimum temperature (Tmin, $^\circ$C) from weeks 13 (late March) to 52 (late December). Soil variables include dry bulk density (BDdry, g cm$^{-3}$), clay percentage (clay, %), soil pH (pH), drained upper limit (dul, mm mm$^{-1}$), soil saturated hydraulic conductivity (ksat, mm day$^{-1}$), wilting point (ll, mm mm$^{-1}$), soil organic matter (om, %), sand percentage (sand, %), and saturated volumetric water content (sat, mm mm$^{-1}$) at nine different depths of soil: 0–5, 5–10, 10–15, 15–30, 30–45, 45–60, 60–80, 80–100, and 100–120 cm. Weather data and Soil data were available at 1 km$^2$ spatial resolution. To compute county-level information, we had to scale up and aggregate the soil and weather information. We took the average of soil at different spatial resolutions at a county to compute county-level soil information. In contrast, We took the median of weather at different spatial resolutions at a county to scale up the county-level weather information. Management variables include acres planted at the county-level, weekly cumulative percentage of planted and harvested acreages. We also created additional variables using the weather and management data based on agronomic insight to help enhance the performance of the model, such as growing degree days, number of rainy days, and heat units. Due to the lack of publicly available genotypic data, we extracted two new variables using additional data from the National Agricultural Statistics Service^34^ to account for the trend of genetic improvements^2^: (1) trend of historical yields and (2) trend of population density for corn and pod count for soybean. These two variables were put in the category of management variables. All variables were normalized to the [0, 1] interval.

### Step 2: Robust feature and interaction selection

To avoid overfitting, we selected a subset of all explanatory variables (features) to predict crop yield. We applied elastic net regularization model to select a set of high-quality features for each category of weather, soil, and management, and then we used forward and backward stepwise selection to identify features and interaction that are spatially and temporally robust across different counties over different years. These robust features and interactions were selected using a similar algorithm from our previous study^35^, which was modified to iterate between exploring new interactions and cross-validating their performances. Such process continues until a set of robust features and interactions has been discovered that lead to good prediction accuracy on the training data and generalizability on the validation data. The way interactions were represented in our model differs from the classical factorial interaction. However, they are also similar in the sense that our algorithm explores all possible factorial combinations to identify the most effect interactions to include in the model.

### Step 3: Linear regression

The last step of the prediction model is a multiple linear regression, which attributes crop yield to additive contributions from weather, soil, management, and their interactions. As such, this prediction model combines the strengths of explainability of linear regression, prediction accuracy of machine learning, and agronomic insights.

More details about the kernel functions in Eq. (1) and the algorithm for solving it are provided in Appendix 1.

### Experimental setting

We compared the performance of the proposed algorithm with that of eight other machine learning algorithms from the literature: linear regression was implemented in R; stepwise regression was implemented in R using the MASS package^36^; LASSO, ridge, and elastic net were implemented in R using the glmnet package^37^; random forest was implemented in R using the ranger package^38^; extreme gradient boosting (XGBoost) was implemented in R using the xgboost package^39^; and neural network was implemented in Python using the Sklearn package^40^. We fed all original explanatory variables as input to these eight algorithms. The linear regression algorithm uses all features without interaction selection; stepwise regression, Lasso regression, ridge regression, and elastic net have their default feature selection settings in the software packages without interaction selection; random forecast, xgboost, and neural network use different modeling structures for feature and interaction selection. As such, the different performances of these algorithms can be attributed to how they select features and interactions from the same set of explanatory data.

All nine algorithms were deployed to predict both corn and soybean yields in the states of Illinois, Indiana, and Iowa from 2015 to 2018. To predict yield for the test year *t*, the training data included all the explanatory (weather, soil, and management) and response (crop yield) data from 1990 to year $t-1$. A 10-fold CV over training and validation partitions was applied to tune the hyperparameters using a grid search approach.

**Figure 2:**
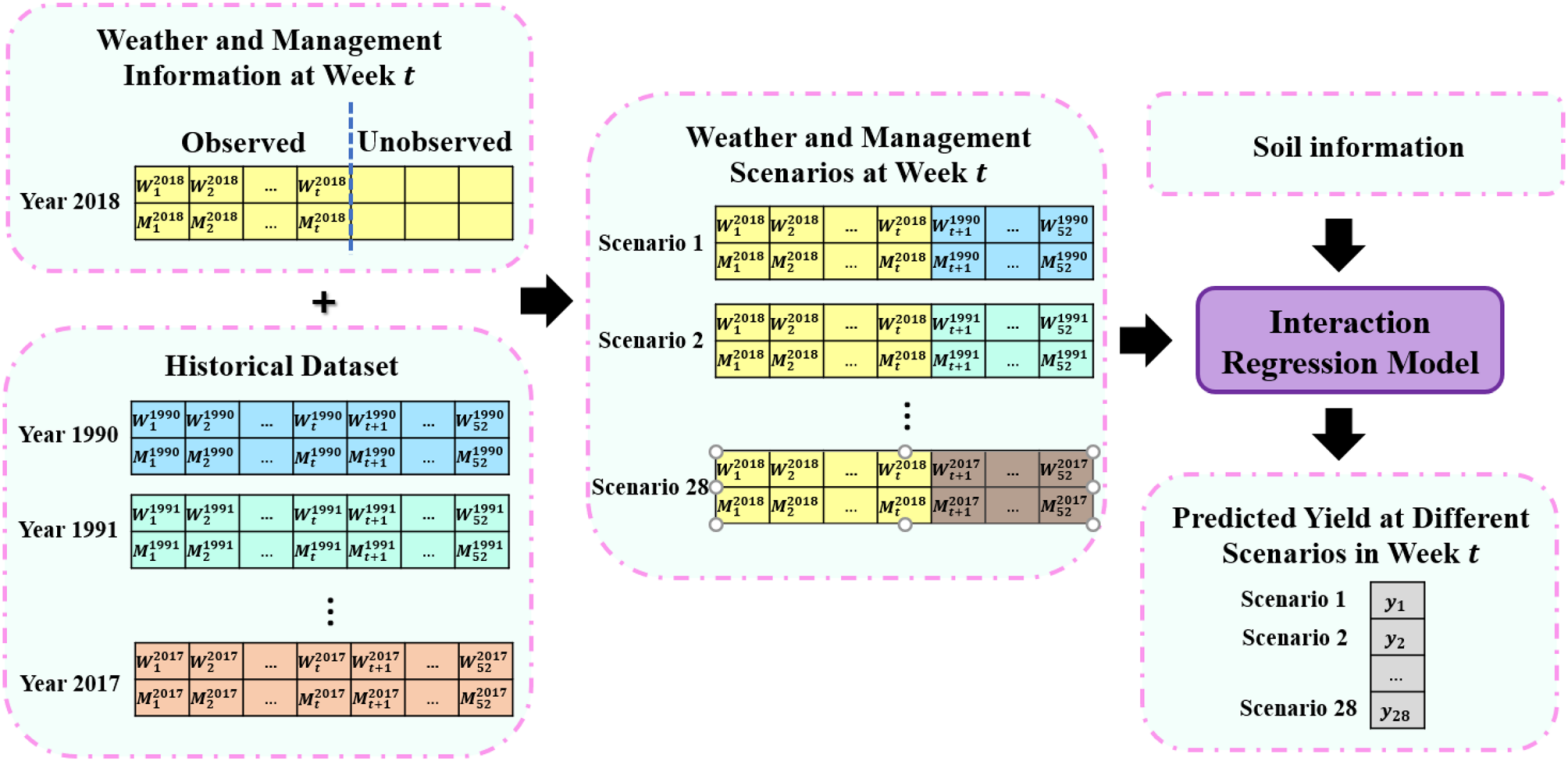
Illustration of generating scenarios and predicting yield at each week during the growing season. This plot was created with Microsof PowerPoint (Version 16.0.12827.20200 32-bit).

Crop yield prediction during the growing season is informative for farmers to make economic or management decisions, but it is also very challenging due to weather and management uncertainty. Our model was able to provide weekly predictions by integrating continuously updated weather and management data with future weather scenarios. For this purpose, first, we trained the proposed model for historical information, and then we utilized this trained model to predict yield performance during the growing season. The process of generating scenarios during growing season and predicting yield performance was illustrated in Fig. 2. For the prediction at each week, we recorded observed weather and management information and estimated them in advance to construct the whole weather and management profiles. For unknown part of data, we used the observed ones from previous years as different scenarios at each week. Therefore, we could generate several predictions for corn and soybean for each week corresponding to each scenario. By observing more and more weather and management data, the uncertainty decreased; thus, the prediction accuracy was expected to improve over time as more actual observations by being available to replace estimated weather and management. Our previous work using a crop model suggested that weather uncertainty decreased by 60% by mid-July in Iowa for both corn and soybean^41^. The final prediction at each week was the median of yield performances of scenarios.

To explore the prediction performance of the proposed Interaction–Regression model for corn and soybean in complete unseen counties, we created four datasets by removing the historical dataset of some counties from the training and validation sets. For the first three datasets, we removed data for Illinois (IL), Indiana (IN), and Iowa (IA) from training and validation sets, respectively; for the last dataset, we randomly picked 100 out of the 293 counties and removed all their data from training and validation sets. For this purpose, for the test dataset of unseen counties in 2018, the historical dataset of seen counties from 1990 to 2017 was divided into four time-wise folds. Then, the proposed framework used these folds for feature selection and interaction detection. After extracting robust features and interactions for each dataset, we partitioned validation and training sets as two previous years from the test year 2018 (years 2016 and 2017) and dataset corresponding to the rest of the years to 1990 (years 1990 to 2015), respectively. Then, for each test dataset, we trained the model using its training partition and robust features and interactions, and the trained models were utilized to predict crop yield of the unseen counties in the year 2018.

## Results

### Prediction accuracy comparison with other machine learning models

Prediction errors for two crops over four test years using nine algorithms are summarized in Table 1. More comparison in terms of the relative RMSE (RRMSE), the relative squared error (RSE), the mean absolute error (MAE), the relative absolute error (RAE), and the coefficient of determination ($R^2$) of nine models are reported in Appendix 2. These results suggested that the proposed model outperformed other models for all test years for both corn and soybean in all evaluation criteria. The test root mean square errors (RMSE) are also lower than what has been reported in the literature^13,14,16,29^. As such, the different performances of our model and others can be attributed to how our model selects high-quality and robust features and interactions from the same set of explanatory data. Second, due to the sparsity of the modeling structure by specifically separating interactive effects from additive effects of features, the algorithms are less prone to overfitting than some machine learning approaches. In terms of the computation time, the proposed approach took approximately two hours for each test year, which was comparable with the neural network model.

**Table 1:** RMSE (in t/ha) of nine algorithms for corn and soybean yield prediction over four test years.

Model	Corn test year				Soybean test year			
	2015	2016	2017	2018	2015	2016	2017	2018
Linear regression	1.39	1.33	1.19	0.96	0.52	0.48	0.42	0.43
Stepwise regression	1.37	1.13	1.16	0.97	0.42	0.34	0.35	0.36
Lasso regression	1.41	1.31	1.21	0.92	0.42	0.42	0.31	0.31
Ridge regression	1.32	1.29	0.99	0.95	0.41	0.43	0.34	0.32
Elastic net	1.25	1.26	1.03	0.93	0.40	0.40	0.32	0.33
Random forest	1.30	1.20	1.06	0.94	0.34	0.37	0.28	0.39
XGBoost	1.50	1.37	1.24	1.08	0.43	0.46	0.40	0.44
Neural network	1.24	0.82	0.95	0.93	0.40	0.37	0.31	0.40
Interaction regression	1.02	0.81	0.90	0.81	0.29	0.27	0.23	0.27

### Prediction performance with known weather after growing season

**Figure 3:**
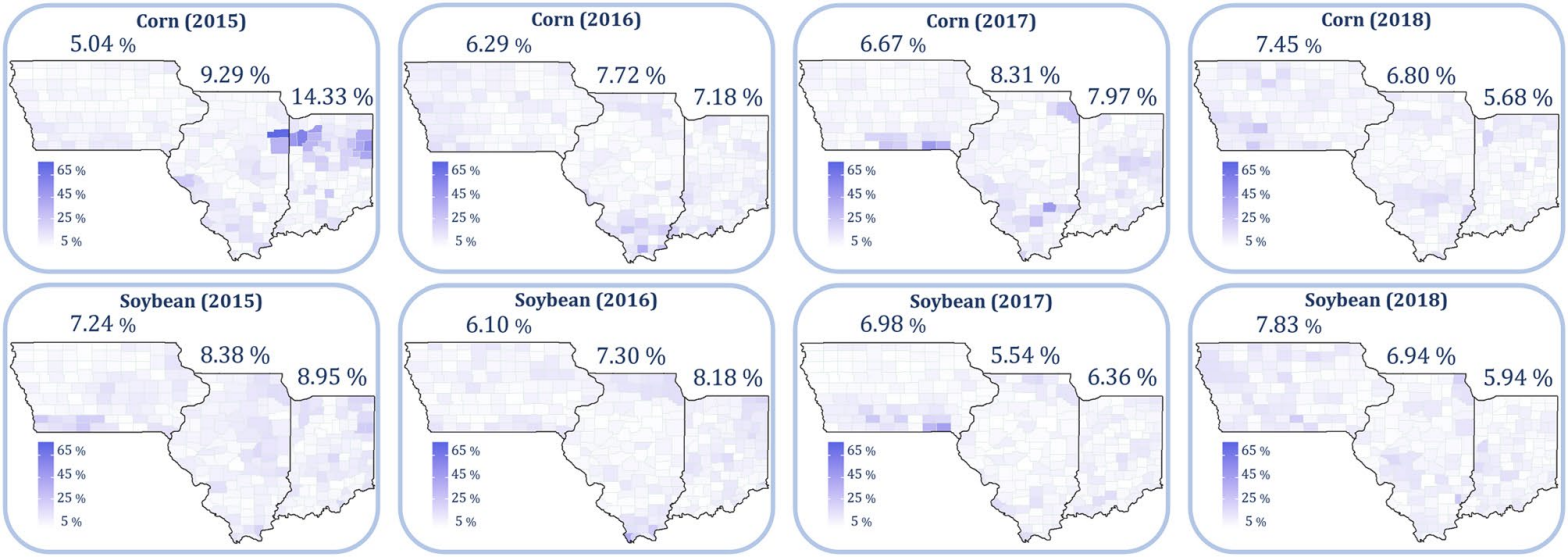
RRMSE for corn and soybean yield prediction from 2015 to 2018. These plots were created with R (version 3.6.3)^43^.

Figure 3 illustrates the prediction performance of the proposed model after the end of the growing season when all the weather data have been observed. These results indicate that the proposed model has an RRMSE lower than 8% in all three states (and most of the counties) over multiple years for both corn and soybean. In reference, prediction accuracy of other recent studies ranged from 7.6% mean absolute percentage error for corn using deep neural networks^42^ to 16.7% RRMSE for corn using random forest^8^.

### Prediction performance with updating weather during growing season

**Figure 4:**
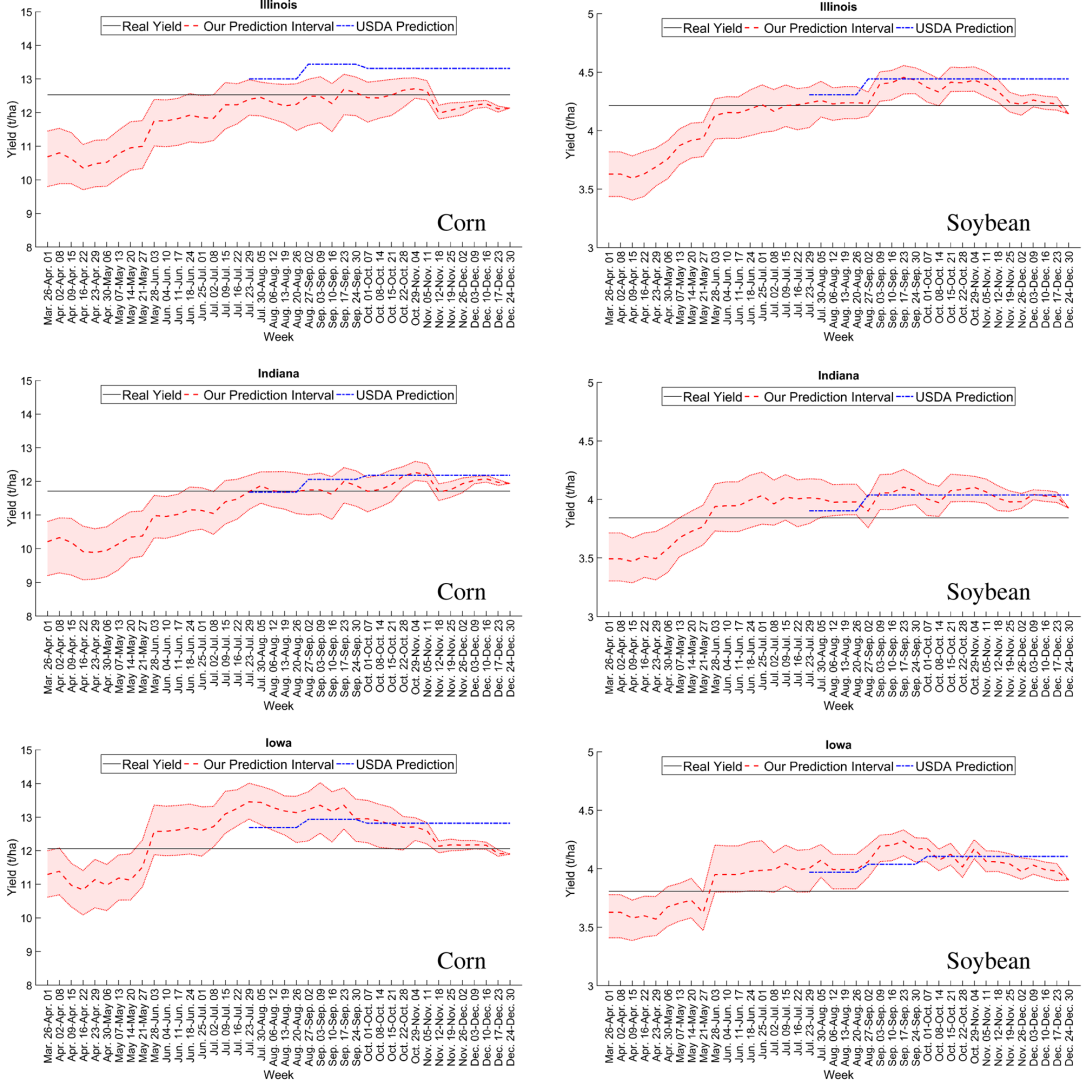
State-level predictions of corn and soybean during the growing season for three states in 2018. Our model provided weekly predictions based on observed weather information; prediction intervals were constructed using historical weather scenarios for yet-to-be-observed weather. The dashed red curve is the median prediction, and the pink interval is defined by the first and third quantiles under multiple weather scenarios, constructed using historical weather data. The dotted blue curves are USDA predictions, which were released in August, September, and October of 2018 at the state level. The solid black line indicates the actual state average yield, which was announced by USDA in February 2019. These plots were created with MATLAB R2018a (Version 9.4.0.813654 64-bit).

Figure 4 shows the predictions of corn and soybean yield during the growing season of 2018 in the three states, updated weekly to incorporate new weather data. Compared with the USDA predictions, results from the proposed model have two advantages: (1) interval predictions throughout growing season with weekly updates, (2) county level (as opposed to state level) predictions with well accuracy. The pattern of increased yield prediction from April to July was caused by weather and planting time in 2018, and it varied across different counties. Our prediction continues to update until the end of December, which is more than 2 months after the end of the growing season. This is because the model is able to capture factors that affect crop yield from crop maturity to harvest, such as adverse weather conditions during harvesting.

### Temporal and spatial extrapolation performance

The prediction performance of the proposed Interaction–Regression model for corn and soybean in unseen counties at the test year 2018 are reported in Table 2. Investigation on the performance of the proposed model using four datasets by removing the historical dataset of some counties from the training and validation sets suggest that the proposed approach has a satisfactory prediction performance in both temporal and spatial extrapolation.

The result of corn yield prediction reveals that a trained model using two selected states from Illinois, Indiana, and Iowa is able to predict corn yield at selected states with at most 8.98 % error. In contrast, soybean prediction of unseen locations using a trained model of seen locations cannot provide robust enough soybean yield prediction. It means that corn yield is more predictable than soybean yield at completely unseen locations with new weather, soil, and management profiles. Also, results suggest that soybean yield prediction is more sensitive to the model compared with corn yield.

**Table 2:** RMSE in t/ha (and RRMSE in %) of the interaction regression model for the extrapolation of crop yield for unseen counties at the year 2018.

Crop	Training and validation sets	Test set	Training RMSE (RRMSE)	Validation RMSE (RRMSE)	Test RMSE (RRMSE)
Corn	IA and IN	IA and IN	0.56 (6.19%)	1.20 (10.3%)	1.52 (12.82%)
		IL			0.83 (6.67%)
	IA and IL	IA and IL	0.60 (6.61%)	0.82 (6.80%)	1.15 (9.37%)
		IN			0.79 (6.79%)
	IL and IN	IL and IN	0.59 (6.75%)	0.66 (5.93%)	0.71 (5.90%)
		IA			1.08 (8.98%)
	193 random counties	193 random counties	0.62 (6.85%)	0.68 (5.89%)	0.75 (6.23%)
		The other 100 counties			0.75 (6.30%)
Soybean	IA and IN	IA and IN	0.19 (6.51%)	0.20 (5.42%)	0.30 (7.86%)
		IL			0.37 (8.94%)
	IA and IL	IA and IL	0.19 (6.54%)	0.18 (4.81%)	0.30 (7.55%)
		IN			0.64 (16.77%)
	IL and IN	IL and IN	0.20 (6.87%)	0.18 (4.97%)	0.24 (6.09%)
		IA			0.85 (22.47%)
	193 random counties	193 random counties	0.20 (6.95%)	0.18 (4.96%)	0.30 (7.71%)
		The other 100 counties			0.29 (7.39%)

Each row shows the dataset by removing all historical information of counties. The rest of the dataset was partitioned into validation and training sets as two previous years from the test year 2018 (years 2016 and 2017) and dataset corresponding to the rest of the years to 1990 (years 1990–2015). First test set refers to the prediction of counties with historical datasets in training and validation set at the test year 2018 (temporal extrapolation). Second test set refers to the prediction of unseen counties with no historical dataset in training and validation set at the test year 2018 (temporal and spatial extrapolation).

## Explainable insights

**Figure 5:**
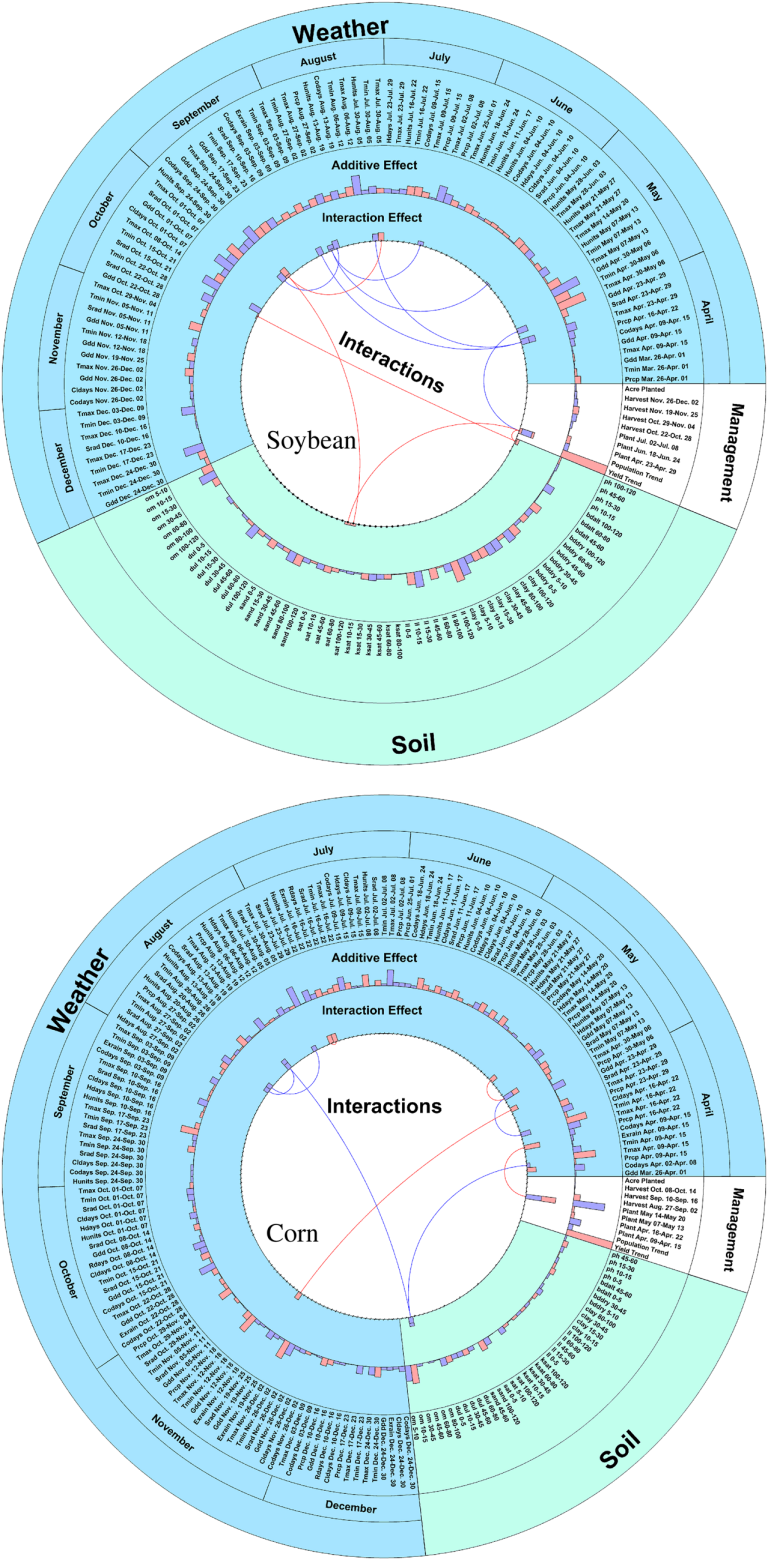
The circular graphs indicate additive and interactive effects for corn and soybean. Curves inside the inner circle connect the two variables involved in the two-way interactions. The bars in the first layer around the circle represent the effects of the interactions, and the bars in the second layer show the additive effects of the features. Positive and negative effects are illustrated with red and blue colors, respectively. These plots were created with MATLAB R2018a (Version 9.4.0.813654 64-bit).

The proposed model provided accurate predictions and some additive and interactive effects, which could help farmers, breeders, and agronomists better understand the complex and interactive relationship among environment and management. Our model selected 202 robust features and 11 two-way interactions to predict the corn yield. Out of the 202 features, 155 were for weather, 37 for soil, and 10 for management. In reference, the total number of variables is 613 (including 440 for weather, 90 for soil, 83 for management), thus the total number of possible two-way interactions is $613^2 = 375,769$ (quadratic effects are considered self-interactions^44,45^). These features and interactions were carefully selected to balance prediction accuracy with spatial and temporal consistency. As such, the same set of features and interactions apply to all counties in the three states for all years between 2015 and 2018. Similarly, our model selected 160 robust features (including 91 for weather, 59 for soil, and 10 for management) and 12 two-way interactions to predict the soybean yield. The contributions of the selected features and interactions for corn and soybean are visualized in Fig. 5.

**Figure 6:**
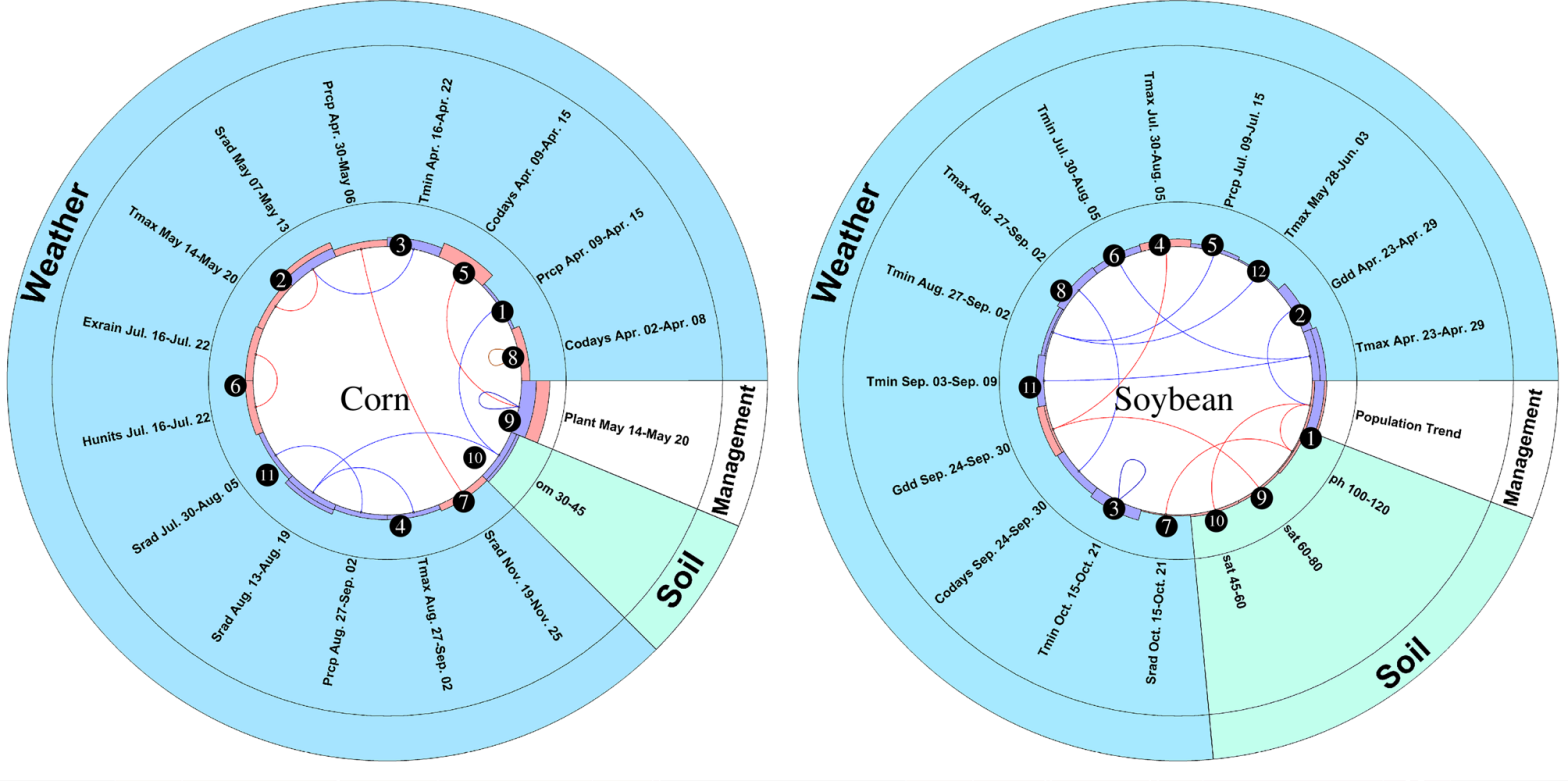
The circular graphs show that interactions for corn (left) and soybean (right) that were discovered by the proposed model. Curves inside the inner circle connect the two variables involved in the interactions. The first layer outside the circle shows the positive (red) or negative (blue) effects of the interactions. These plots were created with MATLAB R2018a (Version 9.4.0.813654 64-bit).

The size of the bars shows the effects of variables and interactions on yield performance. The yield trend indicates a significant factor in estimating the yield of both corn and soybean. Soybean has one self-interactions which includes minimum temperature between October 15 and October 21, and it has negative effects on soybean yield. Corn has two self-interactions, including cold days from April 2 to April 8 and cumulative percentage of planted acreages from May 14 to May 20 with positive and negative effects, respectively. The number of weather factors in estimating corn yield is more than soybean yield. In contrast, the number of soil factors in estimating soybean yield is more than twice the number of soil factors in the prediction of corn yield. Corn yield is more sensitive than soybean yield to management factors. Detected interactions reveal that most of the interactions are between weathers from April to September (emergence to reproductive stages). Moreover, temperature plays an important role in most interactions as maximum and minimum temperature and numbers of cold days. A close-up view of the interactions are shown in Fig. 6 in two lower circular graphs, in which all 11 interactions for corn and 12 for soybean are numbered.

**Figure 7:**
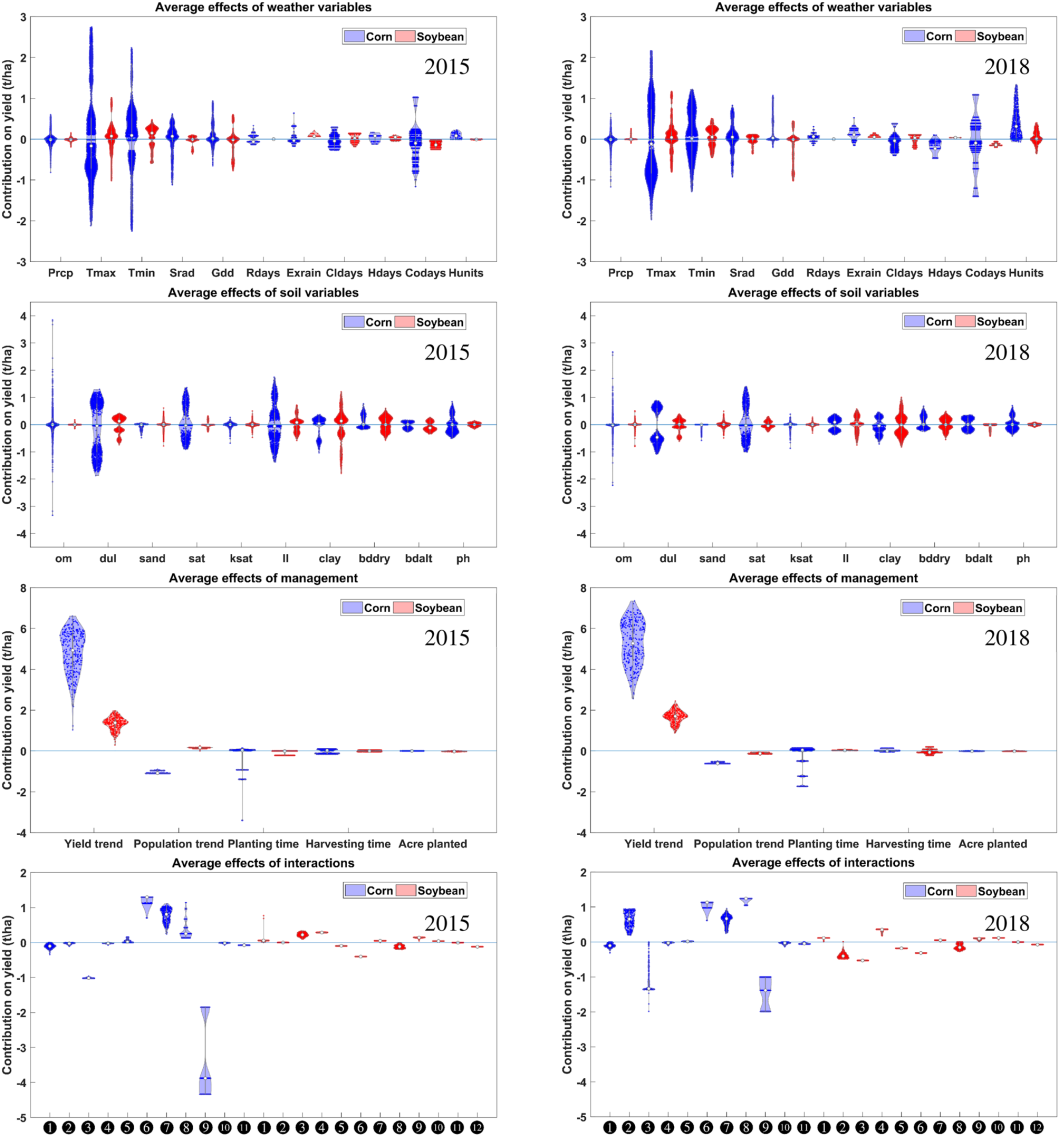
Violin plots of estimated contributions of weather (first row), soil (second row), management (third row) and interaction (fourth row) variables on corn and soybean yield in 2015 (left) and 2018 (right). Each dot on a violin plot represents a county level observation. X-axis numbers of lower panels correspond to the associated numbers to interactions in Fig. 6. These plots were created with MATLAB R2018a (Version 9.4.0.813654 64-bit).

We explain the contributions of weather ($\beta _\text {W} W$), soil ($\beta _\text {S} S$), management ($\beta _\text {M} M$), and their interactions ($\beta _\text {I} I$) in all counties in 2015 and 2018 as violin plots in Fig. 7. The size of the violin plot is denoted as the contribution of parameters to yield. Although their contributions are changed from year to year, high-impact features, including maximum and minimum temperatures, number of cold days, soil organic matter, wilting point, planting time, and yield trend show high contributions to yield continuously over time. The skewness of the yield trend and heat units contributions are on the positive side, which means they increase yield performance. High-variance in temperature, soil organic matter, wilting point, clay percentage, and drained upper limit indicate that the counties across the US Corn Belt have experienced very different climates and have wide soil structures, especially in 2015. Cumulative percentage of planted acreages as the self-interaction ninth in corn yield prediction negatively impacts yield performance at most $-4.5$ t/ha in 2015 and $-2$ t/ha in 2018. However, interactions number 6, 7, and 8 contribute positively to corn yield. Interactions play an important role in the yield prediction of corn compared with soybean. Results also reveal that weather conditions in earlier weeks of the growing season have more influences on yield than later ones, and that late planting time is associated with lower yield performance. These findings are consistent with results from field experimental studies^41,46–51^.

### Insightful interactions

**Figure 8:**
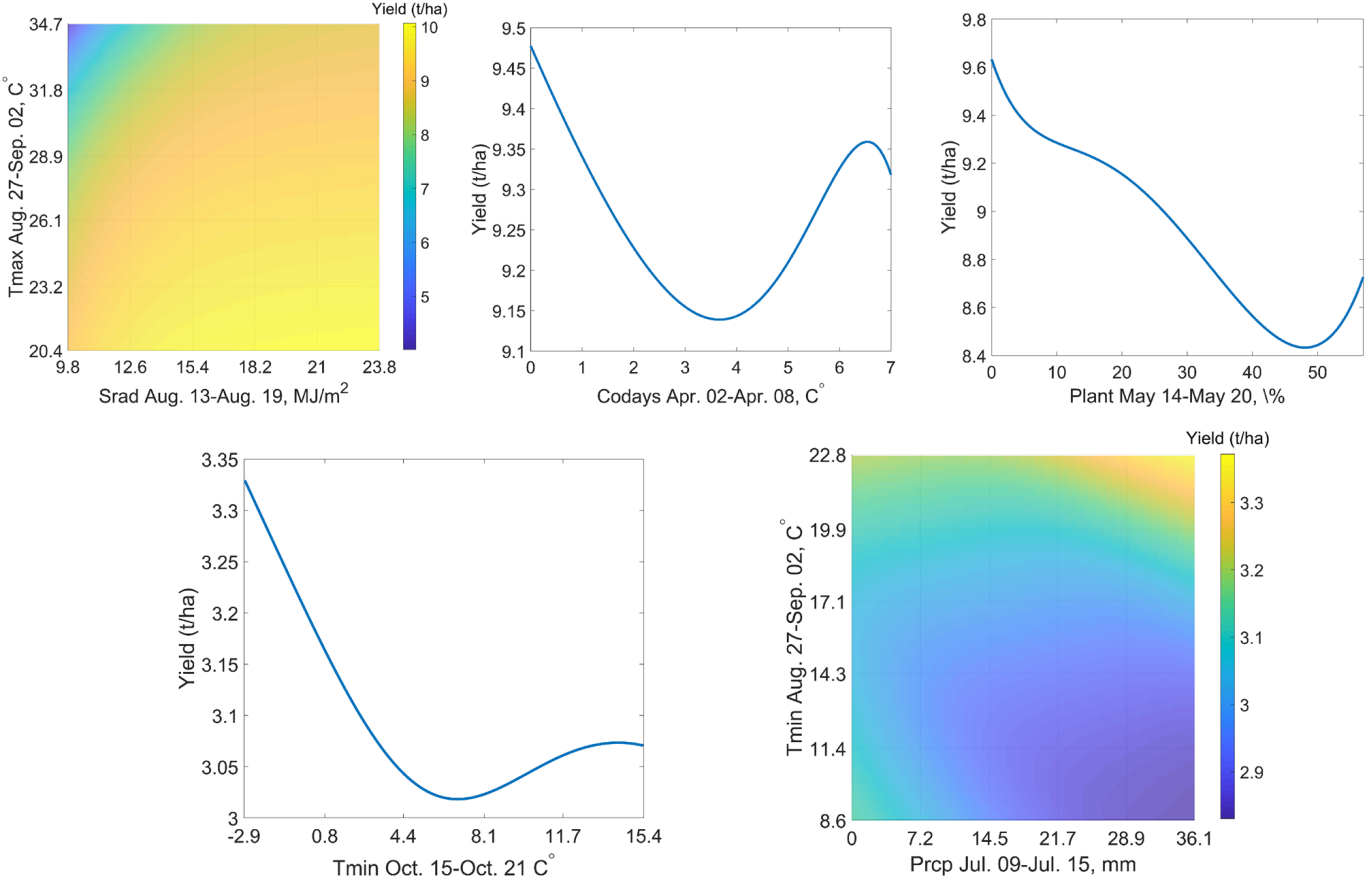
The upper row indicates partial dependence plots of interactions ❹ (left), ❽ (center), and ❾ (right) for corn. The lower row shows partial dependence of interactions ❸ (left) and ❺ (right) for soybean. These plots were created with MATLAB R2018a (Version 9.4.0.813654 64-bit).

The upper row of Fig. 8 illustrates three of the interactions for corn using partial dependence plots, which is a popular way to show the marginal effect that one or two features have on the predicted outcome of a machine learning model.

- Two-way interaction ❹ for corn: the combination of low solar radiation and high maximum temperature during the late grain filling period negatively affects corn yields. This is consistent with agronomic intuition, as low solar radiation limits the energy for photosynthesis, and high maximum temperatures are associated with additional yield losses through tissue respiration and increased evapotranspiration stress.
- Self interaction ❽ for corn: average yield drops from 9.455 to 9.15 t/ha as the number of cold days in the week of April 2 increases from 0 to 4. This is insightful because the soil organic matter mineralization and soil water evaporation will slow down in low temperature, leading to delayed field operations due to reduced production of nitrogen and wetter soil surface. The upward trend of yield as the number of cold days increases from 4 to 7 days is counter-intuitive biologically, but it may reveal an important agronomic insight: when the low temperatures last long enough, farmers may start to take actions (e.g., more fertilization and irrigation) to offset its negative impact on corn yield.
- Self interaction ❾ for corn: completing planting by May 14 is ideal for the yield, and leaving 50% of planting unfinished by May 20 may reduce the yield by 1.25 t/ha. This is consistent with the well-known benefit of early planting^47^. It was also validated in 2019, when the weather-caused delay in planting in IL and IN led to decreased yields^34^.

The lower row of Fig. 8 illustrates two of the interactions for soybean using partial dependence plots.

- Self interaction ❸ for soybean: lower temperature, even near freezing, in mid- to late-October is favorable for soybean yield.
- Two-way interaction ❺ for soybean: high precipitation in mid July makes the yield sensitive to night temperature in late August; warmer nights may lead to a 0.45 t/ha higher yield than cooler nights. It has been reported that higher temperature will negatively impact soybean yield^52,53^; our results further suggest that precipitation may also affect the extent of such impact. A possible interpretation is that higher temperature accelerates leaf senescence and increases remobilization of nitrogen and dry matter from vegetative tissues to grains, and such process may be more sensitive to temperature at a higher level of soil moisture.

### Dissection of crop yield

**Figure 9:**
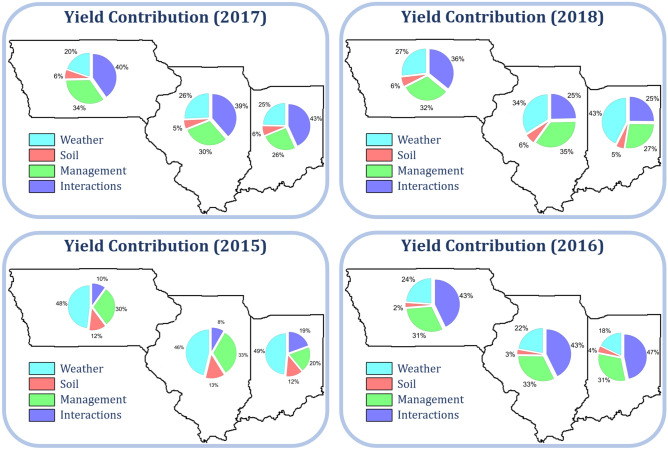
Breakdown of observed corn yield in three states from 2015 to 2018 to contributions of weather ($\beta _\text {W} W$), soil ($\beta _\text {S} S$), management ($\beta _\text {M} M$), and their interactions ($\beta _\text {I} I$). These plots were created with R (version 3.6.3)^43^.

**Figure 10:**
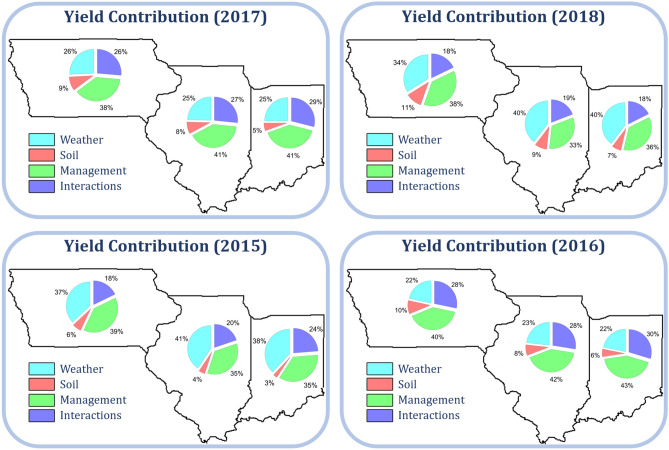
Breakdown of observed soybean yield in three states from 2015 to 2018 to contributions of weather ($\beta _\text {W} W$), soil ($\beta _\text {S} S$), management ($\beta _\text {M} M$), and their interactions ($\beta _\text {I} I$). These plots were created with R (version 3.6.3)^43^.

Breakdowns of observed yields in three states from 2015 to 2018 to contributions of weather ($\beta _\text {W} W$), soil ($\beta _\text {S} S$), management ($\beta _\text {M} M$), and their interactions ($\beta _\text {I} I$) are shown in Figs. 9 and 10 for corn and soybean, respectively. These contributions differ by county and change over time. In 2015, weather was the deciding variable for the yield, whereas interactions played a more important role in 2018. Due to the relatively static nature and lack of dramatic changes across the three Midwest states, soil variables demonstrated a lower effect on crop yield than the dynamic weather, management, and their interactions^28,54^.

The main contributions of the proposed model are summarized in its three salient properties compared with other machine learning models.

The first property is to use robust features and interaction for designing a yield prediction model from year to year prediction. From an agronomic point of view, the conventional feature selection techniques are not proper for yield prediction due to changing train data set from year to year leads to a selection of a different set of features. Hence, the biological results from a different set of features are different. The lack of this robust selection structure is felt.

Second, the proposed model addresses the limitation of machine learning models in transparency by deciphering environment by management interactions for corn and soybean yield. The proposed model was designed efficiently to select a subset of interactions spatially and temporally to result in high performance and less prone to the overfitting problem.

Third, The proposed model quantifies contributions of weather ($\beta _\text {W} W$), soil ($\beta _\text {S} S$), management ($\beta _\text {M} M$), and their interactions ($\beta _\text {I} I$) to observed yield, where capable machine learning models such as neural network, random forest, and XGBoost cannot quantify these contributions.

## Conclusion

We proposed the interaction regression model for crop yield prediction, which made three major contributions. First, it outperformed state-of-the-art machine learning algorithms with respect to prediction accuracy in a comprehensive case study, which used historical data of three Midwest states from 1990 to 2018. Second, it was able to identify about a dozen E $\times$ M interactions for corn and soybean yield, which are spatially and temporally robust and can be used to form counter-intuitive, insightful, and testable hypotheses. Third, it was able to explain the contributions of weather, soil, management, and their interactions to crop yield. Achieving these three contributions simultaneous is particularly significant, since no other crop yield prediction algorithms have been able to satisfactorily address both prediction accuracy and explainability.

The proposed model and computational experiments are not without limitations. For example, the robust feature and interaction selection algorithms were heuristic in nature, which can find high-quality solutions efficiently but do not guarantee global optimality. By increasing the number of features (genetic information), the proposed heuristic algorithm maybe lose its efficiency in terms of running time in finding robust features and interactions. Our model is seeking self -or two-way interactions. New models are required to discover high-order interactions between variables. The non-linear functions of interaction in this paper are limited to six defined kernel functions that can be extended in future research. The performance of the algorithm may be further improved by applying more advanced techniques for hyperparameter tuning^55^. Due to lack of publicly available information on genotype and management, the W, S, and M data used in our case study may be disproportional to their true contributions to crop yield. However, the proposed modeling approach was designed for both discrete and continuous explanatory variables and capable of analyzing all G, W, S, and M variables and their interactions. Future research should explore the possibility of including additional data (such as high-dimensional genotype data, plant traits, detailed management strategies, and satellite images) to further improve prediction accuracy and make more biologically and agronomically insightful discoveries.

## Supplementary Information

Supplementary Information 1.

## Data Availability

The implementation of the proposed model and dataset used in this study are available at https://github.com/ansarifar/An-Explainable-Model-for-Crop-Yield-Prediction.
