# Supplementary material for: An interaction regression model for crop yield prediction
Source: Sci Rep. 2021 Sep 7;11:17754. doi: 10.1038/s41598-021-97221-7 (PMC8423743; doi:10.1038/s41598-021-97221-7)
Supplement: Supplementary file 1 — Supplementary Information 1. [file 41598_2021_97221_MOESM1_ESM.pdf]

An Explainable Model for Crop Yield Prediction

Javad Ansarifar[1,*], Lizhi Wang[1], and Sotirios V. Archontoulis[2]

[1]Department of Industrial and Manufacturing Systems Engineering, Iowa State University, Ames, IA 50011, USA
[2]Department of Agronomy, Iowa State University, Ames, IA 50011, USA
[*]ansarifar.javad@gmail.com

Appendix 1: Details of Prediction Model

The prediction model can be formulated in Equation (1) as follows:

$$\hat{y}_i = \beta_0 + \sum_{j\in\mathscr{P}} X_{i,j}\beta_j + \sum_{m\in\mathscr{M}} b_m Z_{i,m}, \quad \forall i \in \mathscr{N}. \tag{1}$$

Key to model element in Equation (1) is to decipher the interaction matrix Z from the input data. In this paper, we use a kernel-based approach to represent the interactions as

$$Z_{i,m} = \sum_{k\in\mathscr{K}} \delta_{m,k} K_k(r_{i,m}(X_i, \alpha_m)),$$

where vector $\alpha_m \in \{0, 0.5, 1\}^{|\mathscr{P}|}$ indicates variables that trigger the interaction m; $r_{i,m}$ defines relative difference between involved variables in interactions m of county i; $K(\cdot)$ is the kernel function; and where $\delta_{m,k}$ is a binary variable indicating whether interaction m is best described by the type k kernel ($\delta_{m,k} = 1$) or not ($\delta_{m,k} = 0$). The proposed model specify the best type of kernel by satisfying the constraint $\sum_{k\in\mathscr{K}} \delta_{m,k} = 1$. Vector α_m specifies which variables trigger interaction m by assigning one value among three options $\{0, 0.5, 1\}$ to $\mathscr{P}$ variables. If $\alpha_{m,j} = 0.5$, then variable j is not involved in interaction m. If $\alpha_{m,j} \neq 0$, then variable j trigger interaction m. We define the interactions such that, the proposed model is able to capture the non-linear self-effects of variables (self-interaction) as well as two-way interactions between variables on yield. Therefore, $r_{i,m}$ of two-way interaction between two variables j and l ($\sum_{j\in\mathscr{P}} |\alpha_{m,j} - 0.5| = 1, \alpha_{m,j} \neq 0.5, \alpha_{m,l} \neq 0.5, j < l$) is defined as

$$r_{i,m}(X_{i,:}, \alpha_m) = (2\alpha_{m,j} - 1)(X_{i,j} + \alpha_{m,j} - 1) + (2\alpha_{m,l} - 1)(-X_{i,l} - \alpha_{m,l} + 1)$$

where the $r_{i,m}$ of self interaction of variable j ($\sum_{j\in\mathscr{P}} |\alpha_{m,j} - 0.5| = 0.5, \alpha_{m,j} \neq 0.5$) is defined as

$$r_{i,m}(X_{i,:}, \alpha_m) = (2\alpha_{m,j} - 1)(X_{i,j} + \alpha_{m,j} - 1)$$

In this research, kernel function $K(\cdot)$ has six possible variants:

$$K_k(r_{i,m}) = \begin{cases} \text{Linear kernel: } r_{i,m}^2 & k=1 \\ \text{Squared exponential kernel: } \sigma_f^2 \exp\left(-\frac{1}{2}\frac{r_{i,m}^2}{\sigma_l^2}\right) & k=2 \\ \text{Exponential kernel: } \sigma_f^2 \exp\left(-\frac{r_{i,m}}{\sigma_l^2}\right) & k=3 \\ \text{Matern 3/2: } \sigma_f^2 \left(1+\frac{\sqrt{3}r_{i,m}}{\sigma_l}\right)\exp\left(-\frac{\sqrt{3}r_{i,m}}{\sigma_l}\right) & k=4 \\ \text{Matern 5/2: } \sigma_f^2 \left(1+\frac{\sqrt{5}r_{i,m}}{\sigma_l}+\frac{\sqrt{5}r_{i,m}^2}{\sigma_l^2}\right)\exp\left(-\frac{\sqrt{5}r_{i,m}}{\sigma_l}\right) & k=5 \\ \text{Rational quadratic kernel: } \sigma_f^2 \left(1+\frac{r_{i,m}^2}{2\theta\sigma_l^2}\right)^{-\theta} & k=6 \end{cases}.$$

Kernel function has three positive-valued parameters: σ_f, σ_l, and θ, which are signal standard deviation, characteristic length scale, and scale-mixture parameters, respectively. The non-linearity of yield in relation to the predictors comes from the kernel function that each interaction has.

Step 1: Data Preprocessing.

We collected weather, soil, management, and yield performance data from publicly available sources for all counties of the states of Illinois, Indiana, and Iowa from 1990 to 2018.

- Weather data were collected from the Iowa Environmental Mesonet[1], which included four daily surface weather parameters at 1 km^2 spatial resolution: precipitation (Prcp, mm), solar radiation (Srad, MJ/m^2), maximum temperature (Tmax, C°), and minimum temperature (Tmin, C°). Weather data from January to March were excluded, and only weeks 13 (late March) to 52 (late December) data were used in the model. We took the median of weather at different spatial resolutions at a county to scale up the county-level weather information.
- Soil data were acquired from the Gridded Soil Survey Geographic Database[2], which included nine parameters at 1 km^2 spatial resolution: dry bulk density (BDdry, g cm^{-3}), clay percentage (clay, %), soil pH (pH), drained upper limit (dul, $mm.mm^{-1}$), soil saturated hydraulic conductivity (ksat, mm/day), wilting point (ll, $mm.mm^{-1}$), organic matter (om, %), sand percentage (sand, %), and saturated volumetric water content (sat, $mm.mm^{-1}$). All of these ten parameters were available at nine different depths of soil: 0-5, 5-10, 10-15, 15-30, 30-45, 45-60, 60-80, 80-100, and 100-120 cm. We took the average of soil at different spatial resolutions at a county to compute county-level soil information.
- Management data were acquired from the National Agricultural Statistics Service[3], which included acres planted at the county-level, the weekly cumulative percentage of planting process and harvested fields at the state-level. More management data, including the weekly cumulative percentage of silking and emerging processes for corn and the weekly cumulative percentage of blooming and emerging processes for soybean, were collected from the National Agricultural Statistics Service[3]. However, we found that these management variables did not improve the prediction accuracy, since Algorithm 1 did not select them as robust features, which is probably because management data are for the state level, whereas yield prediction is at the county level. Due to the lack of publicly available genotype data, we constructed two new features, i.e., the trend of historical yields and trend of population density for corn and pod count for soybean from the National Agricultural Statistics Service[3] to represent the trend of genetic improvements. We combined these features with management data.
- Yield performance data were also acquired from the National Agricultural Statistics Service[3], which included observed average yield performance between 1990 and 2018 for corn and soybean for all 293 counties in the states of Illinois, Indiana, and Iowa.

We also estimated additional features using the weather and management data based on agronomic insight to help enhance the performance of the model. The following weather variables were calculated from the raw weather data and added to the dataset:

- Growing degree days (Gdd, C°), which is $\max\{0, \text{mean(Tmax,Tmin)} - 10\}$, which is a largely used by agronomists and faster to track crop development[4].
- Number of rainy days (Rdays), which defined as the number of days with rain above 5 mm and below 24 mm in a week[5,6].
- Number of extreme rainy days (Exrain), which is the number of days with rain above 24 mm in a week[7].
- Number of heat days (Hdays), which is the number of days with Tmax above 34 C° in a week[8–10].
- Number of cold days (Codays), which is the number of days with Tmin below 5 C° in a week[5,6].
- Number of cloudy days (Cldays), which is the number of days with solar radiation below 10 MJ/m^2 in a week[5,6].
- Heat units (Hunits), which are the summation of $\max\{0, \text{Tmax} - 34\}$ of a week[8–10].

Step 2: Robust Feature and Interaction Selection.

To avoid overfitting, we selected a subset of all explanatory variables (features) to predict crop yield, so that in Equation (**??**) we have $\beta_i = 0$ for all variables that are not selected. We also required the selected features to be spatially and temporally robust across different counties over different years. The performance of our feature selection algorithm was evaluated using a time-wise F-fold (4-fold in our case study) cross validation, as shown in Figure 1. Each fold $f \in \{1,...,F\} = \mathscr{F}$ is corresponding to a particular test year for prediction. For each fold, we considered the partition of data related to two previous years from a test year as the validation set and dataset corresponding to the rest of the years to 1990 as a training set. We denote indices set $\mathscr{N}_f^{\text{Tr}}$, $\mathscr{N}_f^{\text{V}}$, $\mathscr{N}_f^{\text{Te}}$ for training, validation, and test datasets for each fold $f \in \mathscr{F}$.

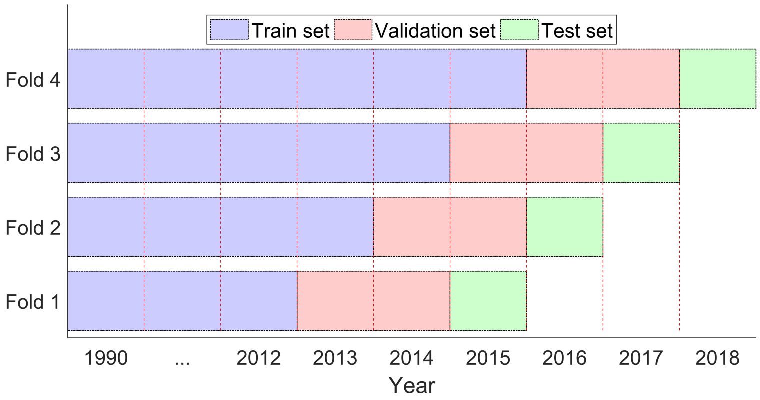

Figure 1. Partition of training, validation, and test datasets for cross-validation.

We cast the prediction problem as the following optimization model. The objective function (2) is our definition of the robustness measure. For any prediction $\hat{y}$ and parameters α and δ, $\zeta_{\text{CV}}^{\text{V}}(\hat{y})$ measures the average RMSE for all F folds of validation datasets. This definition captures temporal and spatial robustness by ensuring, respectively, that the same set of features $\mathscr{P}$ is used for different test years and that the same set of β^f is used for all counties in the same fold.

$$\min \quad \zeta_{\text{CV}}^{\text{V}} = \frac{1}{|\mathscr{F}|}\sum_{f\in\mathscr{F}}\sqrt{\frac{1}{|\mathscr{N}_f^{\text{V}}|}\sum_{i\in\mathscr{N}_f^{\text{V}}}(y_i-\hat{y}_i)^2} \tag{2}$$

$$\text{s.t.} \quad \hat{y}_i = \beta_0^f + \sum_{j\in\mathscr{P}} X_{i,j}\beta_j^f + \sum_{m\in\mathscr{M}} b_m^f Z_{i,m} \qquad i\in\{\mathscr{N}_f^{\text{Tr}},\mathscr{N}_f^{\text{V}}\}, f\in\mathscr{F} \tag{3}$$

$$\begin{bmatrix}\beta_0^f\\ \beta^f\\ b^f\end{bmatrix} = \left[\left(\tilde{X}^f\right)^\top \tilde{X}^f\right]^{-1}(\tilde{X}^f)^\top y \qquad f\in\mathscr{F} \tag{4}$$

$$\tilde{X}_{i,:}^f = [\mathbf{1}, X_{i,:}, Z_{i,:}] \qquad i\in\mathscr{N}_f^{\text{Tr}}, f\in\mathscr{F} \tag{5}$$

$$Z_{i,m} = \sum_{k\in\mathscr{K}} \delta_{m,k}K_k(r_{i,m}(X_i,\alpha_m)) \qquad \forall i\in\mathscr{N}_f, m\in\mathscr{M} \tag{6}$$

$$\sum_{k\in\mathscr{K}} \delta_{m,k} = 1 \qquad \forall m\in\mathscr{M} \tag{7}$$

Model (2)-(7) cannot be solved exactly as a mathematical programming model due to its complex constraints, thus we designed two new algorithms in Step 2 to solve it heuristically, as illustrated in Figure 2. First, the elastic net regularization model[11] is applied to select a set of high-quality features for each fold and each category of soil, weather, and management features. Using the common features of all folds as a starting point, Algorithm 1 and Algorithm 2 are iteratively deployed to find a set of robust features and interactions. Algorithm 1 attempts to improve the robustness measure (2) using a stepwise linear regression approach[12,13] in both backward and forward directions. Algorithm 2 detects interactions among the features identified by Algorithm 1. The interaction of these two algorithms was designed to maximize the robustness measure by balancing validation RMSE and training RMSE. These two algorithms iterate until the termination condition is met, when a set of features and interactions have been found that are temporally and spatially robust. Details of Algorithms 1 and 2 are explained as follows.

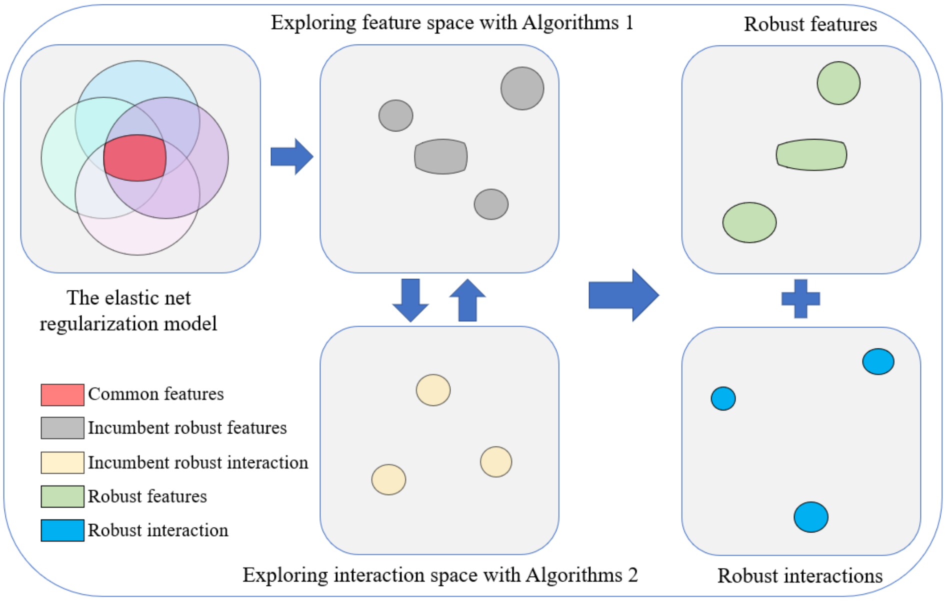

Figure 2. Diagram of step 2. First, the elastic net regularization model is used to select features from each of the folds and use their common features as a starting point for robust features. Then, Algorithm 1 tries to improve the robustness measure (2) using a stepwise linear regression approach in both backward and forward directions, and Algorithm 2 explores potentially significant interactions among these features. The final output of step 2 is a set of features and interactions that are temporally and spatially robust.

Algorithm 1 `Robust feature and interaction selection algorithm`

1: **Input:** Dataset ($X \in [\mathbb{B},\mathbb{R}]^{|\mathscr{N}|\times|\mathscr{P}|}, y \in \mathbb{R}^{|\mathscr{N}|\times 1}$), high-quality features P_f^S, P_f^W, and P_f^M for each fold $f \in \mathscr{F}$.
2: **Output:** Robust feature set $\mathscr{P}^*$ and interactions $\alpha^* \in \{0, 0.5, 1\}^{|\mathscr{M}|\times|\mathscr{P}^*|}$ and their kernel function variable $\delta^* \in \mathbb{B}^{|\mathscr{M}|\times|\mathscr{K}|}$.
3: Initialize robust features set $\mathscr{P}^* = \cap_{f\in\mathscr{F}}(P_f^S \cup P_f^W \cup P_f^M)$ and empty sets j_a and j_r as candidate features for adding and removing, respectively.
4: Apply `Interaction`$(X_{\mathscr{N},\mathscr{P}^*}, y_{\mathscr{N}})$ algorithm to get $\alpha, \delta, \zeta_{\text{CV}}^{\text{Tr}}, \zeta_{\text{CV}}^{\text{V}}$. Initialize $\alpha^* = \alpha, \delta^* = \delta, \zeta_{\text{CV}}^{\text{Tr}*} = \zeta_{\text{CV}}^{\text{Tr}}, \zeta_{\text{CV}}^{\text{V}*} = \zeta_{\text{CV}}^{\text{V}}$.
5: **for each** $j \notin \mathscr{P}^*$ **do**
6: Apply `Interaction`$(X_{\mathscr{N},\mathscr{P}^*\cup j}, y_{\mathscr{N}})$ algorithm to get $\alpha, \delta, \zeta_{\text{CV}}^{\text{Tr}}, \zeta_{\text{CV}}^{\text{V}}$.
7: **if** $\zeta_{\text{CV}}^{\text{V}} < \zeta_{\text{CV}}^{\text{V}*}$ **then**
8: Update $\alpha^* \leftarrow \alpha, \delta^* \leftarrow \delta, \zeta_{\text{CV}}^{\text{Tr}*} \leftarrow \zeta_{\text{CV}}^{\text{Tr}}, \zeta_{\text{CV}}^{\text{V}*} \leftarrow \zeta_{\text{CV}}^{\text{V}}$, and $j_a \leftarrow j$.
9: **end if**
10: **end for**
11: **for each** $j \in \mathscr{P}^*$ **do**
12: Apply `Interaction`$(X_{\mathscr{N},(\mathscr{P}^*\cup j_a)\setminus j}, y_{\mathscr{N}})$ algorithm to get $\alpha, \delta, \zeta_{\text{CV}}^{\text{Tr}}, \zeta_{\text{CV}}^{\text{V}}$.
13: **if** $\zeta_{\text{CV}}^{\text{V}} < \zeta_{\text{CV}}^{\text{V}*}$ **then**
14: Update $\alpha^* \leftarrow \alpha, \delta^* \leftarrow \delta, \zeta_{\text{CV}}^{\text{Tr}*} \leftarrow \zeta_{\text{CV}}^{\text{Tr}}, \zeta_{\text{CV}}^{\text{V}*} \leftarrow \zeta_{\text{CV}}^{\text{V}}$, and $j_r \leftarrow j$.
15: **end if**
16: **end for**
17: **if** $\mathscr{P}^* = (\mathscr{P}^* \cup j_a) \setminus j_r$ **then**
18: **C(1):** Finish.
19: **else**
20: **C(2):** Update $\mathscr{P}^* \leftarrow (\mathscr{P}^* \cup j_a) \setminus j_r$; reset j_a and j_r sets as empty sets, and go to line 5.
21: **end if**

Algorithm 2 `Interaction algorithm`

1: **Input:** Dataset $(X \in [\mathbb{B},\mathbb{R}]^{|\mathscr{N}|\times|\hat{\mathscr{P}}|}, y \in \mathbb{R}^{|\mathscr{N}|\times 1})$, training set $\mathscr{N}_f^{\mathrm{Tr}}$ and validation set $\mathscr{N}_f^{\mathrm{V}}$ for each fold $f \in \mathscr{F}$.

2: **Output:** $\alpha^* \in \{0, 0.5, 1\}^{M\times|\hat{\mathscr{P}}|}, \delta^* \in \mathbb{B}^{M\times|\mathscr{K}|}, \zeta_{\mathrm{CV}}^{\mathrm{Tr}}, \zeta_{\mathrm{CV}}^{\mathrm{V}}$ as local optimal interactions, their kernel function variable, CV training RMSE, and CV validation RMSE, respectively.

3: Initialize the incumbent solution $\alpha^* = 0.5^{1\times|\hat{\mathscr{P}}|}, \delta^* = 0^{1\times|\mathscr{K}|}$, and $M = 1$. Then, go to Step 1.

4: Initialize the current solution as $\hat{\alpha}_{i,j} = \begin{cases} \alpha^*_{m,j}, & \text{if } m \leq M-1 \\ 0.5, & \text{otherwise.} \end{cases}, \forall m \in \{1,...,M\}, j \in \hat{\mathscr{P}}$.

5: Identify the 2-hop neighborhood of $\hat{\alpha}$ as follows.

$$\begin{aligned} \mathscr{A}(M) \;&= \{\alpha \in \{0,0.5,1\}^{M\times|\hat{\mathscr{P}}|} : \|\alpha_{i,:} - \hat{\alpha}_{i,:}\|_0 \leq 2, \forall i \in \{1,...,M\}; \\ &\quad \textstyle\sum_{j\in\hat{\mathscr{P}}} |\alpha_{i,j} - 0.5| \leq 1, \forall i \in \{1,...,M\}\} \end{aligned}$$

6: Evaluate $\zeta_{\mathrm{CV}}^{\mathrm{Tr}} = \zeta(X, y, \mathscr{N}_f^{\mathrm{Tr}}, \mathscr{N}_f^{\mathrm{Tr}}, \alpha, \delta)$ for all $\alpha \in \mathscr{A}(M)$. Let $\bar{\alpha}$ and $\bar{\delta}$ be optimal solutions: $\{\bar{\alpha}, \bar{\delta}\} = \underset{\alpha\in\mathscr{A}(M)}{\arg\min}\, \zeta(X, y, \mathscr{N}_f^{\mathrm{Tr}}, \mathscr{N}_f^{\mathrm{Tr}}, \alpha, \delta)$.

7: **if** $\zeta(X, y, \mathscr{N}_f^{\mathrm{Tr}}, \mathscr{N}_f^{\mathrm{V}}, \bar{\alpha}, \bar{\delta}) < \zeta(X, y, \mathscr{N}_f^{\mathrm{Tr}}, \mathscr{N}_f^{\mathrm{V}}, \alpha^*, \delta^*)$ **then**

8:     **C(1):** Update $\alpha^* \leftarrow \bar{\alpha}$ and $\delta^* \leftarrow \bar{\delta}$; reset $M \leftarrow M+1$, and go to line 4.

9: **else**

10:     **C(2):** Finish.

11: **end if**

The objective of Algorithm 1 is to identify a set of robust features to minimize $\zeta_{\mathrm{CV}}^{\mathrm{Tr}}$, which is the average of training RMSEs. In line 3, the common features of different folds are used as the starting point for the set of robust features. In the two "for" loops, new features are added or removed using a stepwise linear regression approach to further improve the robustness measure $\zeta_{\mathrm{CV}}^{\mathrm{Tr}}$.

The objective of Algorithm 2 is to detect interactions among the features from Algorithm 1 to optimize the robustness measure $\zeta_{\mathrm{CV}}^{\mathrm{Tr}}$. It explores the 2-hop neighborhood of interactions space with all combinations of the kernel functions to optimize the robustness measure. This is an extended version of the algorithm in Ansarifar and Wang (2019)[14] by including not only discrete (genetic) variables but also continues (environment and management) features. This is achieved by normalizing all continues variables to the [0, 1] internal and then using the six kernel functions to capture the potential nonlinear relationship between pairs of features.

Step 3: Linear Regression.

The interaction matrix Z augments the input dataset X with additional features, which helps fit the crop yield with a multiple linear regression model. As such, the model first deploys a powerful optimization engine to identify complex interactions, and then delivers explainable prediction results that can attribute crop yield to additive and interactive contributions of individual explanatory variables.

Appendix 2: Additional Results

Prediction performance of the proposed prediction algorithm for corn and soybean in three states over four test years is reported in Table 1

Table 1. RMSE in t/ha (and RRMSE in %) of the interaction regression model for corn and soybean in three states over four test years.

Crop	Dataset	Test Year 2015	2016	2017	2018
Corn	Train	0.61 (6.97%)	0.62 (6.99%)	0.62 (6.95%)	0.63 (6.99%)
	Validation	1.67 (15.01%)	1.01 (9.08%)	0.92 (8.30%)	0.89 (7.63%)
	Test (3 states)	1.02 (9.60%)	0.81 (7.06%)	0.90 (7.66%)	0.81 (6.73%)
	Test (Illinois)	0.99 (9.29%)	0.88 (7.72%)	0.99 (8.31%)	0.85 (6.80%)
	Test (Indiana)	1.37 (14.33%)	0.75 (7.18%)	0.88 (7.97%)	0.66 (5.68%)
	Test (Iowa)	0.59 (5.04%)	0.79 (6.29%)	0.82 (6.67%)	0.89 (7.45%)
Soybean	Train	0.21 (7.26%)	0.21 (7.26%)	0.21 (7.22%)	0.21 (7.16%)
	Validation	0.27 (8.06%)	0.30 (8.58%)	0.28 (7.75%)	0.26 (7.05%)
	Test (3 states)	0.29 (8.16%)	0.27 (7.18%)	0.23 (6.31%)	0.27 (6.97%)
	Test (Illinois)	0.30 (8.38%)	0.28 (7.30%)	0.20 (5.54%)	0.29 (6.94%)
	Test (Indiana)	0.30 (8.95%)	0.30 (8.18%)	0.22 (6.36%)	0.22 (5.94%)
	Test (Iowa)	0.27 (7.24%)	0.24 (6.10%)	0.26 (6.98%)	0.29 (7.83%)

All nine algorithms were deployed to predict both corn and soybean yields in the states of Illinois, Indiana, and Iowa from 2015 to 2018. To predict yield for the test year t, the training data included all the explanatory (weather, soil, and management) and response (crop yield) data from 1990 to year $t-1$. Cross validation was used to tune hyperparameters for all algorithms. Prediction errors for two crops over four test years using nine algorithms are summarized in Table **??**. Prediction comparisons in terms of the relative RMSE (RRMSE), the relative squared error (RSE), the mean absolute error (MAE), the relative absolute error (RAE), and the coefficient of determination (R^2) of nine models are reported in Tables 2-6, respectively.

Table 2. RRMSE (in %) of nine algorithms for corn and soybean yield prediction over four test years.

Model	Corn Test Year 2015	2016	2017	2018	Soybean Test Year 2015	2016	2017	2018
Linear Regression	13.05	11.57	10.13	8.01	14.51	12.53	11.50	10.88
Stepwise Regression	12.86	9.87	9.87	8.05	11.78	8.93	9.68	9.33
Lasso Regression	13.15	11.40	10.25	7.64	11.84	10.89	8.51	8.00
Ridge Regression	12.36	11.22	8.39	7.87	11.46	11.20	9.28	8.21
Elastic Net	11.71	10.96	8.77	7.69	11.32	10.41	8.82	8.57
Random Forest	12.16	10.45	9.03	7.81	9.64	9.66	7.64	10.03
XGBoost	14.06	11.93	10.58	8.99	12.05	11.94	10.89	11.14
Neural Network	11.56	7.12	8.05	7.74	11.28	9.73	8.58	10.10
**Interaction Regression**	**9.60**	**7.06**	**7.66**	**6.73**	**8.16**	**7.18**	**6.31**	**6.97**

Table 3. RSE of nine algorithms for corn and soybean yield prediction over four test years.

Model	Corn Test Year 2015	2016	2017	2018	Soybean Test Year 2015	2016	2017	2018
Linear Regression	0.86	0.61	0.62	0.49	1.72	1.60	1.10	0.76
Stepwise Regression	0.84	0.44	0.59	0.49	1.13	0.81	0.78	0.56
Lasso Regression	0.88	0.59	0.64	0.44	1.14	1.20	0.60	0.41
Ridge Regression	0.77	0.57	0.43	0.47	1.07	1.27	0.71	0.43
Elastic Net	0.69	0.54	0.46	0.45	1.04	1.10	0.65	0.47
Random Forest	0.75	0.50	0.49	0.46	0.76	0.95	0.48	0.64
XGBoost	1.00	0.65	0.68	0.61	1.18	1.45	0.99	0.79
Neural Network	0.68	0.23	0.39	0.45	1.04	0.96	0.61	0.65
**Interaction Regression**	**0.46**	**0.22**	**0.35**	**0.34**	**0.54**	**0.52**	**0.33**	**0.31**

Table 4. MAE (in t/ha) of nine algorithms for corn and soybean yield prediction over four test years.

Model	Corn Test Year				Soybean Test Year			
	2015	2016	2017	2018	2015	2016	2017	2018
Linear Regression	1.06	1.07	0.95	0.78	0.42	0.39	0.34	0.34
Stepwise Regression	1.11	0.91	0.94	0.79	0.34	0.28	0.28	0.29
Lasso Regression	1.08	1.06	0.96	0.74	0.34	0.33	0.24	0.25
Ridge Regression	1.05	1.06	0.76	0.77	0.32	0.35	0.27	0.25
Elastic Net	0.95	1.02	0.79	0.74	0.32	0.33	0.25	0.26
Random Forest	0.98	0.95	0.84	0.76	0.28	0.29	0.22	0.31
XGBoost	1.20	1.09	0.98	0.85	0.34	0.37	0.33	0.36
Neural Network	1.01	0.66	0.72	0.73	0.32	0.30	0.25	0.32
**Interaction Regression**	**0.74**	**0.66**	**0.69**	**0.65**	**0.23**	**0.22**	**0.17**	**0.22**

Table 5. RAE of nine algorithms for corn and soybean yield prediction over four test years.

Model	Corn Test Year				Soybean Test Year			
	2015	2016	2017	2018	2015	2016	2017	2018
Linear Regression	0.86	0.75	0.78	0.68	1.28	1.34	1.03	0.88
Stepwise Regression	0.90	0.64	0.77	0.70	1.05	0.96	0.87	0.74
Lasso Regression	0.87	0.75	0.79	0.65	1.05	1.15	0.75	0.66
Ridge Regression	0.85	0.74	0.62	0.68	0.98	1.21	0.81	0.64
Elastic Net	0.77	0.72	0.65	0.65	0.97	1.13	0.77	0.67
Random Forest	0.80	0.67	0.68	0.67	0.85	1.01	0.67	0.81
XGBoost	0.97	0.77	0.80	0.75	1.05	1.27	1.00	0.93
Neural Network	0.82	0.46	0.59	0.65	0.97	1.03	0.77	0.83
**Interaction Regression**	**0.60**	**0.46**	**0.56**	**0.58**	**0.71**	**0.76**	**0.53**	**0.56**

Table 6. R^2 of nine algorithms for corn and soybean yield prediction over four test years.

Model	Corn Test Year				Soybean Test Year			
	2015	2016	2017	2018	2015	2016	2017	2018
Linear Regression	0.13	0.38	0.37	0.50	-0.72	-0.60	-0.10	0.23
Stepwise Regression	0.15	0.55	0.40	0.50	-0.13	0.18	0.21	0.43
Lasso Regression	0.11	0.40	0.35	0.55	-0.14	-0.20	0.39	0.58
Ridge Regression	0.22	0.42	0.56	0.52	-0.07	-0.27	0.28	0.56
Elastic Net	0.30	0.45	0.53	0.54	-0.04	-0.10	0.34	0.52
Random Forest	0.24	0.49	0.50	0.53	0.23	0.04	0.51	0.35
XGBoost	-0.01	0.34	0.31	0.38	-0.18	-0.45	0.01	0.20
Neural Network	0.31	0.76	0.60	0.54	-0.04	0.03	0.38	0.34
**Interaction Regression**	**0.53**	**0.77**	**0.64**	**0.65**	**0.45**	**0.47**	**0.66**	**0.68**

Figure 3 provides complementary information to Figure **??** on the estimated contributions of weather and soil to crop yield.

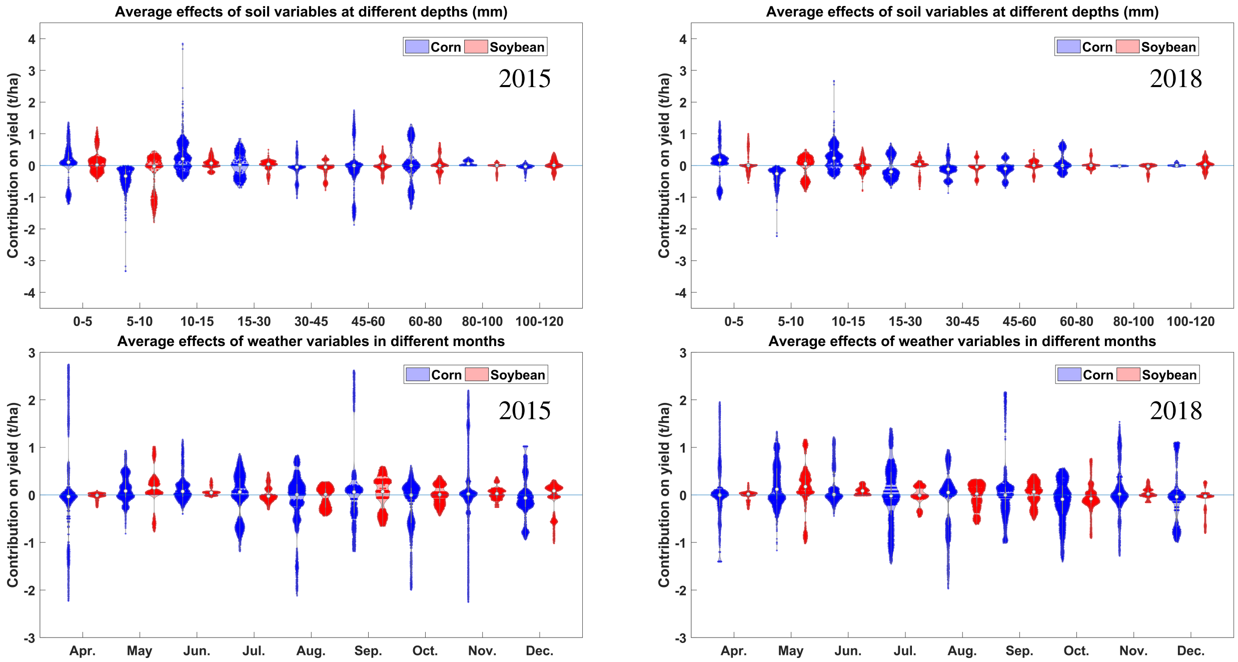

Figure 3. Violin plots of estimated contributions of soil variables (top) and weather variables (bottom) on corn and soybean yield in 2015 (left) and 2018 (right). Each dot on a violin plot represents a county level observation.

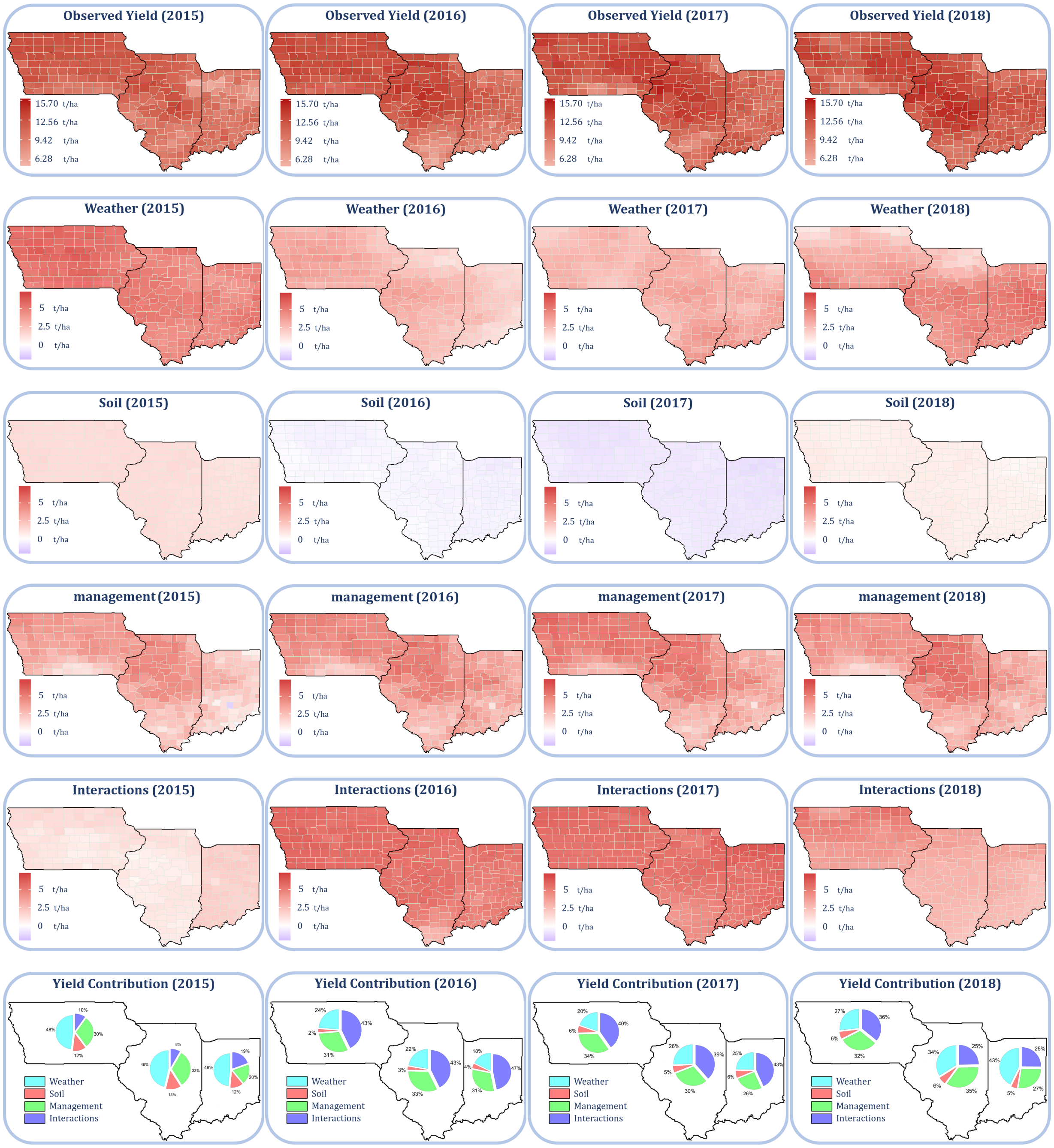

Figure 4. Breakdown of observed corn yield in three states from 2015 to 2018 to contributions of weather ($\beta_W W$), soil ($\beta_S S$), management ($\beta_M M$), and their interactions ($\beta_I I$).

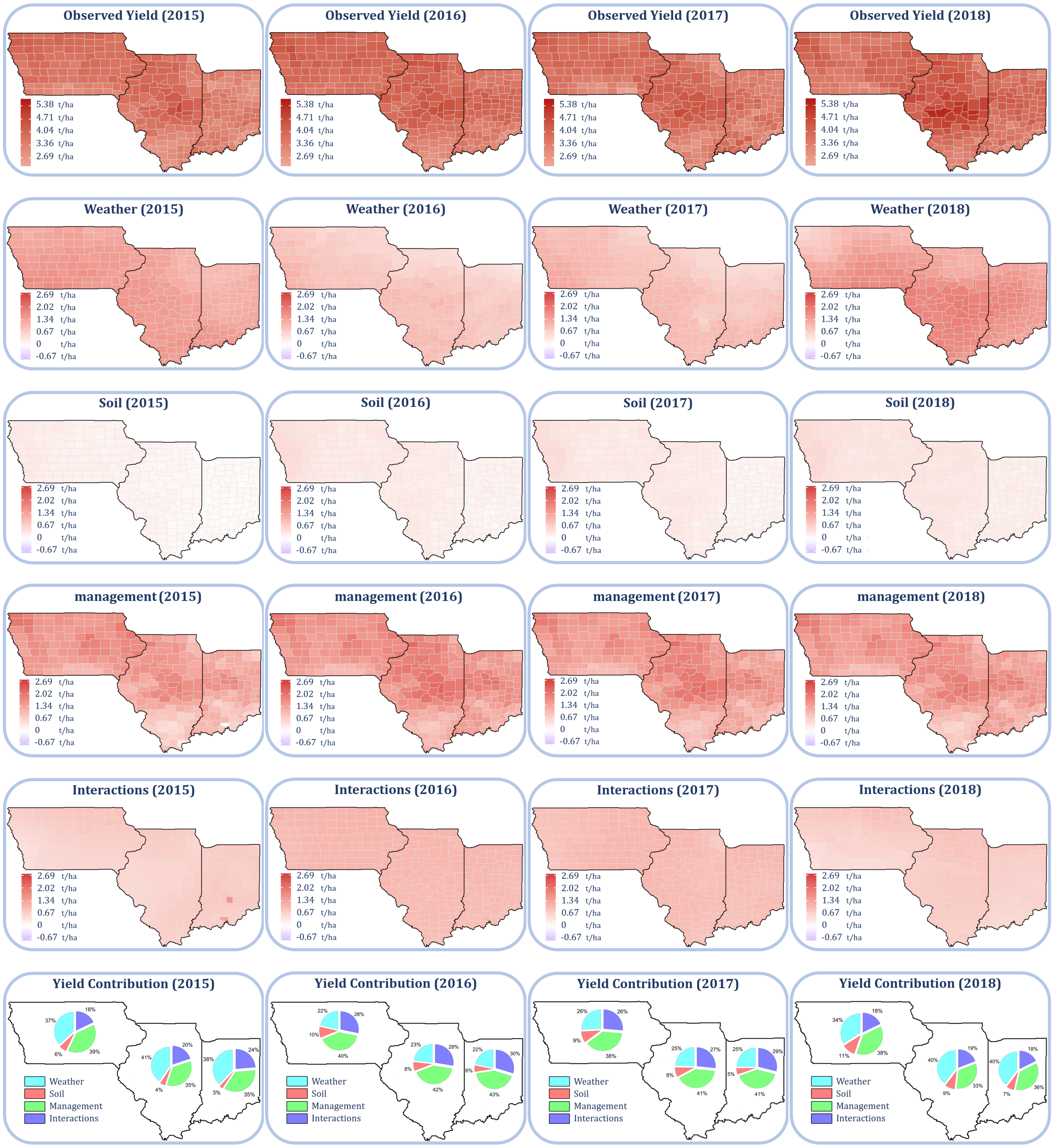

Figure 5. Breakdown of observed soybean yield in three states from 2015 to 2018 to contributions of weather ($\beta_W W$), soil ($\beta_S S$), management ($\beta_M M$), and their interactions ($\beta_I I$).

References

1. Environmental Mesonet, I. https://mesonet.agron.iastate.edu.
2. Database, G. S. S. G. https://gdg.sc.egov.usda.gov.
3. Service, N. A. S. https://quickstats.nass.usda.gov.
4. Abendroth, L. J., Elmore, R. W., Boyer, M. J. & Marlay, S. K. Corn growth and development (2011).
5. Walsh, M. K. *et al.* Climate indicators for agriculture. *USDA Tech. Bull. 1953* 1–70 (2020).

6. Forecast & of Cropping sysTemS (FACTS), A. https://crops.extension.iastate.edu/facts/weather-tool.
7. Puntel, L. A., Pagani, A. & Archontoulis, S. V. Development of a nitrogen recommendation tool for corn considering static and dynamic variables. *Eur. J. Agron.* **105**, 189–199 (2019).
8. Hawkins, E. *et al.* Increasing influence of heat stress on french maize yields from the 1960s to the 2030s. *Glob. Chang. Biol.* **19**, 937–947 (2013).
9. Schlenker, W. & Roberts, M. J. Nonlinear temperature effects indicate severe damages to us crop yields under climate change. *Proc. Natl. Acad. Sci.* **106**, 15594–15598 (2009).
10. Schauberger, B. *et al.* Consistent negative response of us crops to high temperatures in observations and crop models. *Nat. Commun.* **8**, 1–9 (2017).
11. Zou, H. & Hastie, T. Regularization and variable selection via the elastic net. *J. Royal Stat. Soc. Ser. B (Statistical Methodol.* **67**, 301–320 (2005).
12. Venables, W. N. & Ripley, B. D. *Modern applied statistics with S-PLUS* (Springer Science & Business Media, 2013).
13. Hastie, T. J. & Pregibon, D. Generalized linear models. In *Statistical Models in S*, 195–247 (Routledge, 2017).
14. Ansarifar, J. & Wang, L. New algorithms for detecting multi-effect and multi-way epistatic interactions. *Bioinformatics* **35**, 5078–5085 (2019).
